# Photoisomeric Molecule-Mediated Ion Anchoring and UV Resistance in Metal Halide Perovskites

**DOI:** 10.34133/research.1196

**Published:** 2026-03-18

**Authors:** Wenying Zhao, Duo Qu, Yongli Zhang, Chuanzhen Shang, Xuewen Zhang, Chenyun Wang, Bin Zhou, Jingyuan Qiao, Ruilin Han, Shasha Wang, Yuyao Bi, Siyu Wei, Zheng Bao, Fan Xu, Fengjun Ye, Yongguang Tu

**Affiliations:** ^1^ State Key Laboratory of Flexible Electronics (LOFE) and Institute of Flexible Electronics (IFE), Shaanxi Key Laboratory of Flexible Electronics, MIIT Key Laboratory of Flexible Electronics (KLOFE), Northwestern Polytechnical University, Xi’an 710072, China.; ^2^ Harbin Institute of Technology, No. 92 Xidazhi Street, Nangang District, Harbin City, Heilongjiang Province 150001, China.; ^3^ Shanghai Institute of Space Power-Sources, No. 2965 Dongchuan Road, Minhang District, Shanghai 200245, China.; ^4^ Beijing Solarverse Optoelectronic Technology Co., Ltd, Beijing 100176, China.; ^5^ Shenzhen Institute for Advanced Study, University of Electronic Science and Technology of China, Shenzhen 518110, China.

## Abstract

Halide perovskites are novel photovoltaic materials with soft ionic lattice that are continuously exposed to sunlight during operation. High-energy ultraviolet (UV) irradiation will cause halide ion oxidation within the perovskite layer, accelerating halide ion migration and component loss, which results in a marked degradation in the device’s photovoltaic performance. Here, we introduce a 2,3-bis(2,4,5-trimethyl-3-thienyl) maleimide (BTTM) molecule capable of interconversion between UV and visible light into the perovskite structure, intensifying the light stability of the perovskite layer via ion anchoring. Meanwhile, the incorporation of BTTM promotes the growth of perovskite crystals and efficiently passivates defects within the perovskite film, significantly boosting the open-circuit voltage of the perovskite solar cells. This results in a power conversion efficiency increase from 22.07% to 24.71%. Under UV irradiation (365 nm), BTTM molecules mitigate the degradation of perovskite by suppressing the ion migration of iodide ions. After cumulative exposure to 5 kWh/m^2^ of continuous UV irradiation, BTTM-based devices retain over 90% of their initial power conversion efficiency, demonstrating significantly enhanced UV stability. This study offers a straightforward approach to UV protection, providing novel insights for the environmental application of perovskite solar cells under intense UV irradiation.

## Introduction

As one of the representatives of cutting-edge photovoltaic technologies, since the first report in 2009, perovskite solar cells (PSCs) have garnered widespread attention [1–6]. The outstanding physical characteristics of metal halide perovskite—such as broad absorption spectrum range [7–9], high optical absorption coefficient [10–12], high carrier mobility [12–14], and excellent defect tolerance [15–17]—underpin this growing interest. The power conversion efficiency (PCE) has improved rapidly, with laboratory-scale devices achieving efficiencies exceeding 27.0% [18]. However, compared to traditional crystalline silicon materials, metal halide perovskites exhibit notable inherent instability issue, hindering their large-scale application [19–21]. Notably, due to the unique soft ionic crystal property, perovskite materials are susceptible to photodegradation reactions under strong light irradiation, especially high-energy ultraviolet (UV) irradiation [22–26]. Specifically, I^−^ and organic cations in perovskite will migrate, decompose, volatilize, and even escape from the film [27]. High-energy UV photons will accelerate the reduction of Pb^2+^ and oxidation of halogens, and the perovskite will be further degraded into I_2_ and Pb^0^ [28–31], leading to a sharp decline in photovoltaic performance. The formation of I_2_ means the generation of V_I_ (iodine vacancy) defects within the perovskite crystal structure. This process triggers a cascade reaction: I_2_ combines with I^−^ ions to form I_3_^−^, which further facilitates the migration and loss of halogen components, thereby accelerating the degradation of perovskite [32]. Meanwhile, the resulting Pb^0^ serves as a primary source of deep-level defects, aggravating nonradiative recombination processes and seriously affecting the photovoltaic characteristics and operational robustness of PSCs [33,34]. In addition, photoinduced defects will also decompose the organic transport layer and cause short circuits, forming escape channels for volatile components and leading to irreversible degradation of PSCs [35,36].

Several approaches have been explored in current research to reduce UV photodegradation and enhance the photostability of PSCs, including introduction of luminescent down-conversion materials [37,38], additive engineering for crystal stabilization [39], and application of UV absorption layers [40]. The luminescent down-conversion materials and UV absorption layer methods are fundamentally limited, as they merely shield the device from UV light rather than solving the instability issue of perovskite materials. On the other hand, additive engineering can effectively promote molecular interactions that passivate defects, anchor easily migratory ions, and increase the activation energy for degradation, thereby enhancing the UV photostability of the perovskites and solving the problem at the source [41]. For example, Li et al. [42] incorporated 2-(2-hydroxy-5-methylphenyl) benzotriazole into the perovskite layer, which not only passivated defects and reduced the nonradiative recombination but also absorbed UV light via the reversible ring-opening/closing reactions of its chelate ring, thus markedly improving the UV photostability. Similarly, the UV absorber, 2,2′-dihydroxy-4,4′-dimethoxybenzophenone, was demonstrated to absorb UV radiation through reversible keto-enol tautomerization under continuous intense UV irradiation, thereby delaying their photodegradation process [43]. Song et al. [44] proposed a [2+2] cycloaddition reaction using ethyl 6-bromocoumarin-3-carboxylate for incident UV radiation absorption and to suppress tensile strain, thus improving the efficiency and stability. Furthermore, the plant-derived l-theanine was employed to modulate the crystallization kinetics, successfully inhibiting the UV-induced α-to-δ phase transformation of formamidinium-based perovskite [45]. All the above methods propose additive strategies with both defect passivation effects and UV resistance in PSCs. However, the underlying evolution mechanism of the degradation behavior in perovskite materials under UV irradiation remains insufficiently explored.

Herein, we adopt a simple and efficient additive strategy by incorporating a 2,3-bis(2,4,5-trimethyl-3-thienyl) maleimide (BTTM) molecule into the perovskite. Photoisomerization occurs reversibly in this molecular system, driven by alternating UV and visible light exposure. The addition of BTTM serves to modulate residual stress within the perovskite film and help to inhibit the halogen components, thereby improving the stability of the perovskite lattice robustness and significantly increasing the open-circuit voltage (*Voc*) of PSCs, ultimately elevating the PCE of the device from 22.07% to 24.71%. Furthermore, under UV irradiation, the BTTM molecules can continuously suppress the ion migration of iodide ions through structural tautomerism, alleviating the UV-induced degradation process of the perovskite. After continuous UV irradiation with an irradiance of 5 kWh/m^2^, the BTTM device can still maintain over 90% of its initial PCE, far exceeding the control device that retains only 61.3%. This work provides a straightforward strategy for the long-term UV protection of the PSCs and offers new insights for the application of PSCs in extreme environments with intense UV irradiation.

## Results

**Fig. 1.**
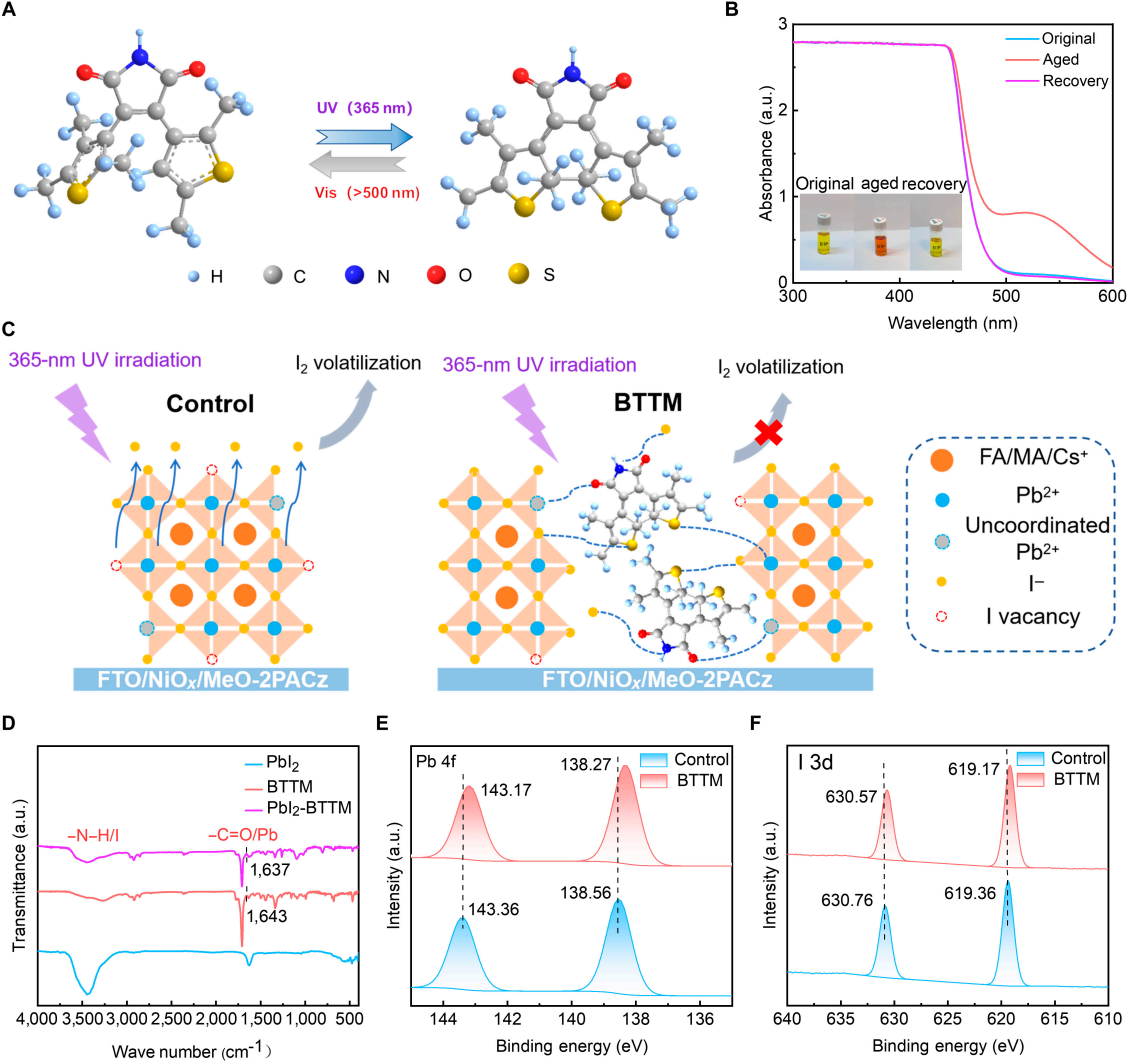
2,3-Bis(2,4,5-trimethyl-3-thienyl) maleimide (BTTM) tautomeric molecules and the interaction between BTTM and perovskite. (A) Illustration of the BTTM molecule. (B) Ultraviolet (UV)–visible absorption spectra of BTTM solution after and before UV aging and recovery. (C) A schematic diagram showing the anchoring effect of BTTM on perovskite under UV light. (D) Fourier transform infrared spectroscopy spectrum of PbI_2_ interacting with BTTM. X-ray photoelectron spectroscopy spectra of the control and BTTM perovskite films: (E) Pb 4f spectrum and (F) I 3d spectrum. a.u., arbitrary units. MA, methylammonium.

As a photoisomeric molecule, BTTM can undergo isomerization between closed- and open-ring forms under UV light and visible light (Fig. 1A) [46,47]. First, we dissolved BTTM in an isopropyl alcohol solution and observed that its color deepened under 365-nm UV light irradiation. As shown in Fig. 1B, the absorption spectrum of the BTTM solution in the UV–visible range (300 to 600 nm) indicates a structural change. Meanwhile, it can be seen that the color of the BTTM solution that has changed after UV aging will return to the state before irradiation after the UV light is removed (inset in Fig. 1B), indicating the reversibility of its tautomeric reaction. Herein, we introduced the BTTM as an additive into the perovskite solution, allowing it to interact with the perovskite. Prior investigations have demonstrated that iodide ions in halide perovskite materials will migrate and form iodine vacancies under UV light irradiation, ultimately leading to the photodegradation of perovskite [48,49]. The C=O functional group in the BTTM molecule engages in a strong bond with Pb in the perovskite. Meanwhile, the N–H group coordinates with I (in the [PbI_6_]^2−^ octahedron) through hydrogen bonding. These interactions will anchor the Pb–I octahedral structure of the perovskite, thereby effectively suppressing the migration of iodide ions and effectively passivating perovskite defects, as shown in Fig. 1C. To verify the interaction of BTTM molecules with iodide ions, we dissolved formamidinium iodide (FAI), BTTM, and FAI+BTTM in isopropyl alcohol, respectively, and irradiated under UV light (365 nm) for different periods of time. As shown in Fig. S1, it can be seen that the color of the FAI+BTTM solution is different from that of the BTTM solution after UV light irradiation. It is speculated that an interaction occurs between FAI and BTTM (Fig. S1B and C). Meanwhile, it can be seen that the FAI solution turned yellow after 4 h of UV light irradiation, indicating that iodide ions escape under UV light irradiation (Fig. S1C). After the FAI+BTTM solution irradiated with UV light for 4 h is placed in an environment without UV light for a period of time, a change in the solution color is also observed, indicating that there is still a certain tautomerism after the interaction between the BTTM and FAI (Fig. S1D). To further verify the interaction between BTTM and the perovskite, we carried out Fourier transform infrared spectroscopy (FTIR) and x-ray photoelectron spectroscopy (XPS) tests. The FTIR results show that the BTTM with –C=O and –N–H groups interacts with FAI and with PbI_2_, as shown in Fig. 1D and Fig. S2. After the interaction between the –C=O in the BTTM and PbI_2_, the observed downward shift of the –C=O symmetric stretching band is from 1,643 to 1,637 cm^−1^, indicating a strong interaction between PbI_2_ and –C=O. Meanwhile, the stretching vibration of N–H also shifts notably, indicating an obvious interaction between the N–H group and I^−^, which further indicates that the BTTM is beneficial for passivating defects in the perovskite film. Figure 1E and F shows the XPS Pb 4f spectra and I 3d spectra of the control and BTTM perovskite films (denoted as control and BTTM, respectively). The XPS spectrum of Pb 4f in the BTTM perovskite film shows that the peak positions of the 2 main peaks, Pb 4f 7/2 and Pb 4f 5/2, are 138.2 and 143.1 eV, respectively, while those of the control perovskite film are located at 138.5 and 143.3 eV. This observation signifies a robust interfacial interaction between the perovskite and BTTM, driven by electron cloud redistribution-induced shifts in binding energy. Meanwhile, the shifts of the I 3d and N 1s peak positions (Fig. S3) further indicate that the N–H group in the BTTM perovskite film interacts with I^−^ to form hydrogen bonds. The results of FTIR and XPS indicate that BTTM would passivate defects in the perovskite film and achieve an anchoring effect on Pb–I, reducing the migration of halide ions, which is expected to improve the photoelectric properties and UV stability of the perovskite film.

Next, we screened the optimal introduction content of BTTM in the perovskite precursor solution. Herein, 0, 0.05, 0.1, 0.25, 0.5, and 1 wt% of BTTM were added to the perovskite precursor solution, respectively. The photos of the films before and after UV light are shown in Fig. S4. It can be seen that the morphology of the 0.1-wt% BTTM film remains relatively good. In addition, to evaluate the impact of BTTM incorporation on film morphology, scanning electron microscopy (SEM) was employed, and images for films with different BTTM contents are displayed in Fig. S5. It can be seen that the perovskite film without BTTM has smaller grains and a large amount of PbI_2_, while the perovskite films with 0.05% and 0.1% BTTM have larger grains and a reduced PbI_2_ content. However, when the added content of BTTM is too high, the morphology of the perovskite film deteriorates, and many voids appear on the surface. Simultaneous x-ray diffraction (XRD) testing (Fig. S6) and time-resolved photoluminescence (PL) testing (Fig. S7) revealed that perovskite films containing 0.1-wt% BTTM exhibited superior crystalline quality and longer carrier lifetime. Compared to the control films, the carrier lifetime increased from 126.41 to 340.82 ns. Finally, we prepared PSCs with varying BTTM concentrations and conducted *J*-*V* testing (Fig. S8). Comparisons revealed that adding BTTM significantly increased *Voc* across all samples, with the 0.1-wt% BTTM group exhibiting that the PCE has been significantly improved. Based on these results, we determined that 0.1 wt% represents the optimal BTTM concentration.

**Fig. 2.**
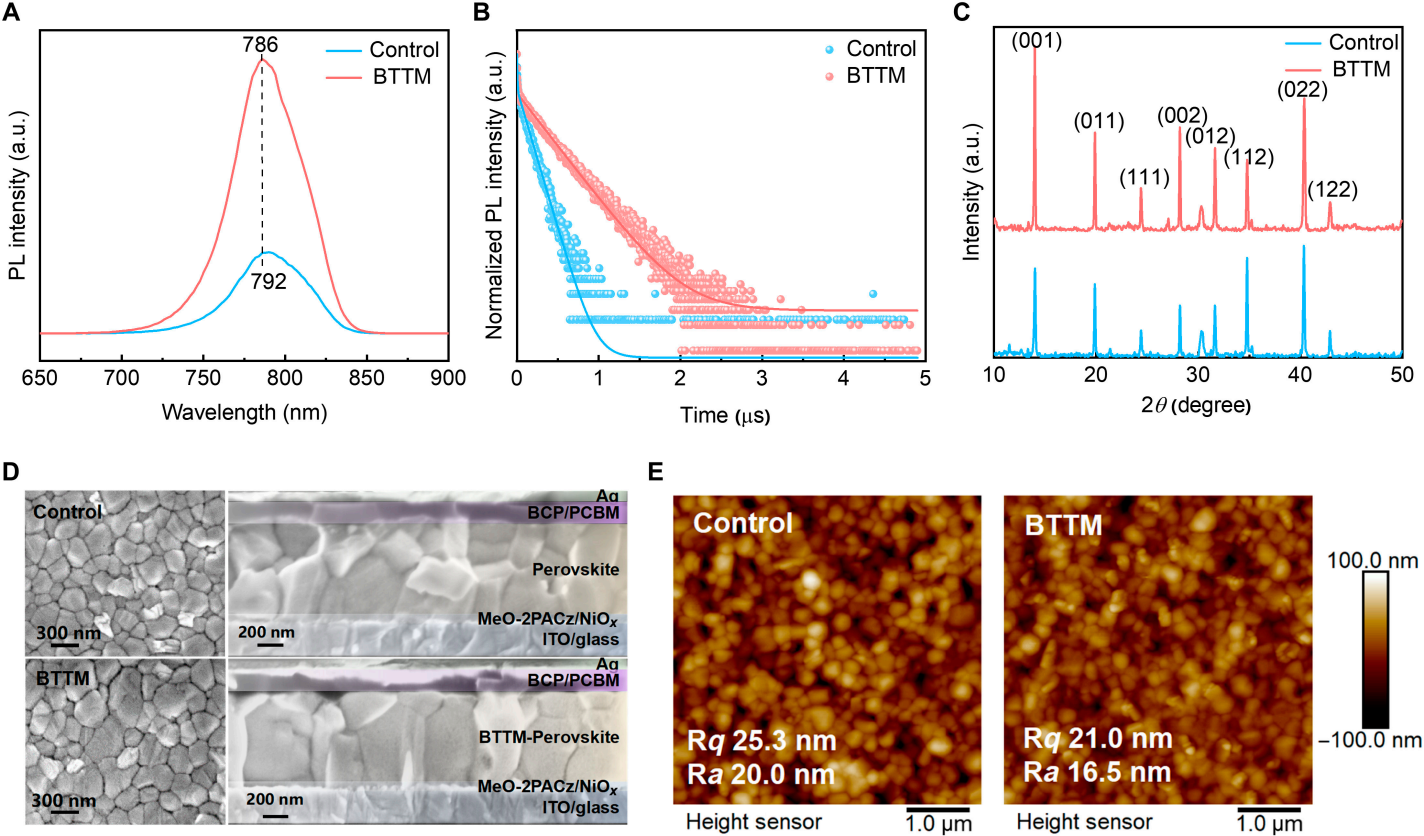
Performance comparison of the control and 2,3-bis(2,4,5-trimethyl-3-thienyl) maleimide (BTTM) perovskite films. (A) Photoluminescence (PL) spectra of the control and BTTM films. (B) Time-resolved PL decay curves of the control and BTTM films. (C) X-ray diffraction (XRD) patterns of the control and BTTM films. (D) Scanning electron microscopy (SEM) images of the upper surface and cross-section of the control and BTTM films. (E) Atomic force microscopy images of the upper surface of the control and BTTM films. a.u., arbitrary units. ITO, indium tin oxide; BCP, bathocuproine; PCBM, (6,6)-phenyl-C61-butyric acid methyl ester.

To assess the impact of BTTM on the carrier dynamics of perovskite films, steady-state PL and time-resolved PL measurements were performed on films from different groups [50]. In subsequent multiple tests, the addition amount of BTTM in the BTTM group was 0.1 wt%. First, in the PL spectrum of the perovskite film (Fig. 2A), it can be seen that the PL intensity is markedly enhanced after adding BTTM. Meanwhile, the PL emission peak of the control film is centered at 792 nm, whereas that of the BTTM-modified film shifts to 786 nm, indicating a 6-nm blueshift relative to the control. This indicates that nonradiative recombination losses are effectively suppressed and that the defects in the film are reduced. Meanwhile, as shown in Fig. 2B, the BTTM film exhibits a longer carrier lifetime than the control film. A longer carrier lifetime means fewer nonradiative recombination paths.

To further investigate the crystallinity and micro-nano morphology of the perovskite films, we compared the XRD patterns of the control and BTTM perovskite (Fig. 2C). Upon introducing BTTM, the diffraction intensity of the (001) plane increased, indicating that BTTM promotes more oriented crystal growth. Notably, the characteristic PbI_2_ peak at 12.7° observed in the control film disappears in the BTTM-treated film, demonstrating that BTTM interacts with PbI_2_ during crystallization and suppresses excess PbI_2_ formation, thereby enhancing film stability. Contact-angle measurements of the pristine and BTTM-modified films (Fig. S9) show only negligible changes, suggesting that BTTM has minimal influence on surface wettability and does not affect subsequent solution-processing steps. Next, SEM and atomic force microscopy analyses were then performed to investigate the impact of BTTM on the micro/nanostructure of the films. [51]. Comparing the surface and cross-sectional SEM images of the control perovskite film (Fig. 2D), it can be seen that after the addition of BTTM, the grain size of perovskite becomes larger and shows a tendency of vertical growth, which is more conducive to the reduction of defects at the grain boundaries and the efficient transport of carriers. Atomic force microscopy images of the control and the BTTM film reveal (Fig. 2E) that the incorporation of BTTM reduces the film’s roughness, with R*q* decreasing from 25.3 to 21.0 nm and R*a* decreasing from 20.0 to 16.5 nm. Residual stress in halide perovskites induces significant lattice distortion, serving as a key factor contributing to material instability [52,53]. To quantify this effect, we conducted grazing-incidence XRD using the 2*θ*-sin^2^*ψ* method, with residual stress determined through linear fitting analysis of the diffraction intensity on the (012) plane (Fig. S10) [54]. The slope decreases from 0.57 (control) to 0.39 (BTTM), indicating that BTTM effectively releases residual tensile stress and modulates the crystallization kinetics of the perovskite film.

**Fig. 3.**
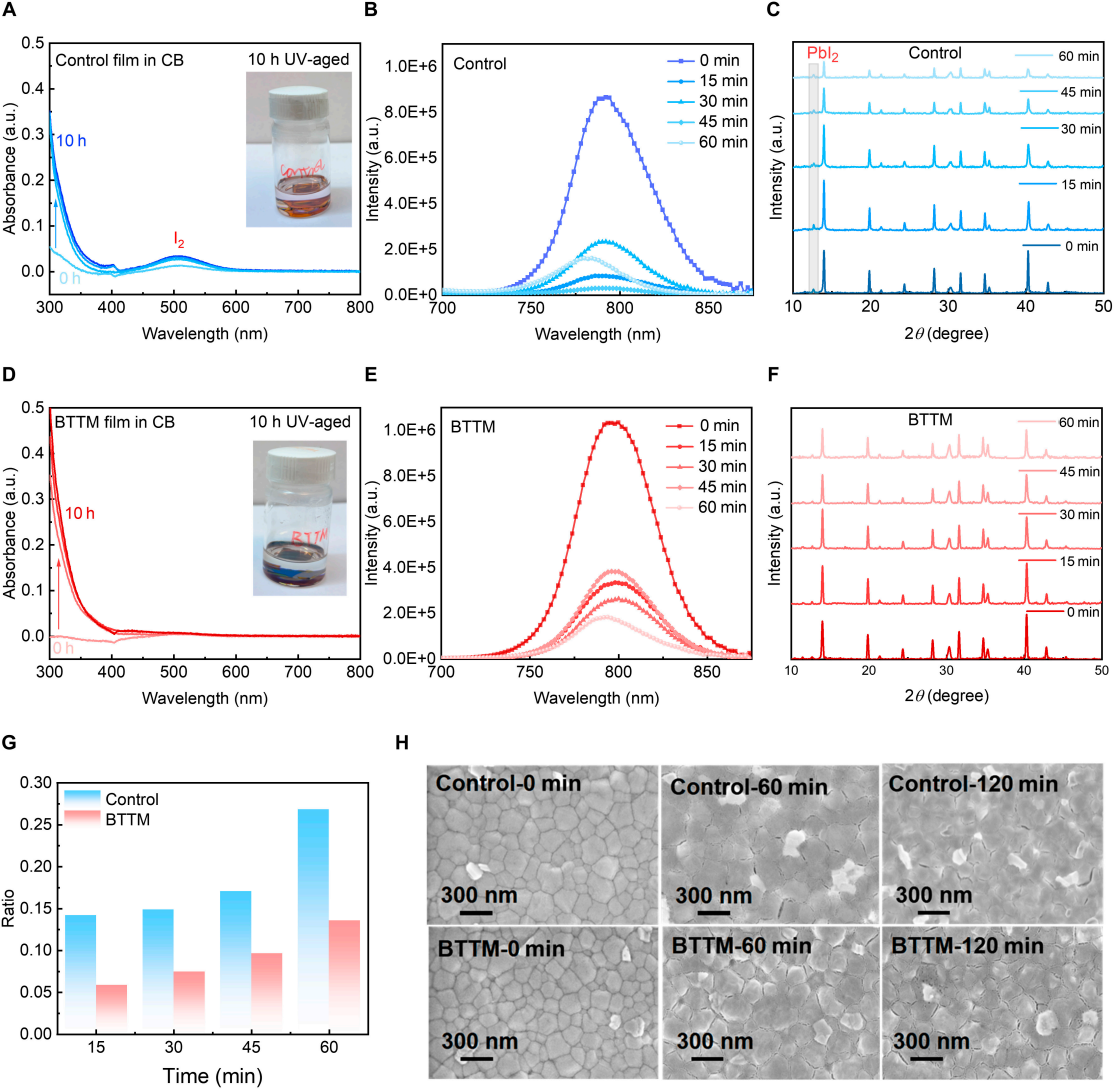
Comparison of performance between control and 2,3-bis(2,4,5-trimethyl-3-thienyl) maleimide (BTTM) perovskite films after ultraviolet (UV) irradiation. (A and D) Absorption spectra of control and BTTM perovskite films immersed in chlorobenzene (CB) under UV light (365 nm) irradiation. (B and E) Photoluminescence spectra of control and BTTM perovskite films under UV irradiation. (C and F) X-ray diffraction patterns of control and BTTM perovskite films under UV light irradiation. (G) Comparison of integral area ratio of PbI_2_ and (001) peak positions with UV irradiation time. (H) Scanning electron microscopy images of control and BTTM perovskite films under UV light irradiation. a.u., arbitrary units.

Based on previous studies, high-energy UV radiation induces the oxidation of halide anions in perovskites. These I_2_ react with I^−^ to form I_3_^−^, followed by ion migration, and this process would lead to progressive degradation and loss of components within the perovskite structure [49,55]. The incorporation of BTTM into perovskite structures enables the absorption of UV radiation and continuous passivation of defects, thereby anchoring I^−^ ions and reducing the production of I_2_ to mitigate UV irradiation damage to perovskite. To validate the anchoring effect of the BTTM on iodine, we immersed both control and BTTM perovskite films in chlorobenzene (CB). It is because CB exhibits low solubility in perovskite films but good solubility in iodine, allowing the degradation reaction of perovskite to be verified by measuring the iodine content in the solution. The samples were then exposed to 365-nm UV irradiation at 200 mW/cm^2^ for 10 h, followed by continuous solution absorbance measurements. The results are shown in Fig. 3A and D. The control film exhibited noticeable color changes, with some samples turning from black to yellow. This transformation likely resulted from the collapse of the perovskite octahedral structure due to iodine ion migration, leading to the loss of A-site organic cations and the formation of PbI_2_. Subsequent UV–visible measurements of the control film’s aged CB solution revealed a distinct I₂ characteristic absorption peak around 500 nm. In contrast, no such specific absorption peak was ever observed in the CB of the BTTM perovskite film. This phenomenon indicates that the introduction of BTTM can reduce the diffusion of iodine in perovskite films during UV aging, which may be due to BTTM stabilizing I^−^, inhibiting its oxidation reaction, and further preventing the photodegradation of perovskite. This directly enhances the stability of the perovskite film components. Additionally, to evaluate ion migration within the thin film, we investigated device behavior at different *J*-*V* scan rates, with particular focus on low scan rates. This is because at slower scan rates, ions have sufficient time to respond to electric field changes, potentially leading to an increase in the hysteresis index (HI) as ion migration activity intensifies. As shown in Fig. S11 and Tables S3 and S4, the HI of the control device increased from 0.009 to 0.014 upon reducing the scan rate. In contrast, the HI of the BTTM device at the low scan rate of 20 mV/s only rose to 0.008—significantly lower than the control device. The observed decrease in HI provides indirect evidence for suppressed ion migration, supporting the “ion-anchoring” hypothesis. For metal halide perovskites, their fluorescence properties can effectively validate and evaluate their optoelectronic performance. We conducted PL testing to examine the evolution of fluorescence characteristics in perovskite films subjected to UV aging, and both control and BTTM perovskite films were exposed to 365-nm UV irradiation at an irradiance of 200 mW/cm^2^. As shown in Fig. 3B and E, the PL intensity of control perovskite films decreases to varying degrees after UV aging. In contrast, BTTM films maintain relatively strong PL intensity even after 1 h of UV irradiation, indicating that the anchoring effect of BTTM preserves the fluorescence properties of perovskite films and reduces defect generation even after UV aging. Additionally, we performed the photoluminescence quantum yield (PLQY) measurements to evaluate nonradiative recombination in perovskite films and tested the films before and after UV aging (Fig. S12). The perovskite film incorporating BTTM exhibited a slight improvement in PLQY compared to the control group. Notably, after 60 min of UV aging at 200 mW/cm^2^, the PLQY of the BTTM film significantly increased to 3.13%, whereas the control film remained at only 1.60%. These results collectively support that nonradiative recombination within the BTTM film has been suppressed.

To verify the stability of perovskite crystals under UV irradiation, we conducted XRD testing on control and BTTM perovskite films exposed to UV light, as shown in Fig. 3C and F. The XRD analysis reveals a substantial decrease in the intensity of diffraction peaks for the control perovskite film as UV exposure time increases. In contrast, the peak intensity of the BTTM perovskite film remains relatively stable, indicating that BTTM effectively mitigates UV-induced structural degradation of the perovskite. Concurrently, as UV irradiation accumulated, a distinct PbI_2_ diffraction peak emerged at 12.7° in the control film. The photostability of this excess PbI_2_ significantly accelerates perovskite degradation, severely compromising crystal stability. To compare the evolution of PbI_2_ generated during decomposition across different film groups over irradiation time, we calculated the integral areas of the PbI_2_ and perovskite (001) crystal plane diffraction peaks. Figure 3G shows the comparison of the ratio of the integrated area of their diffraction peaks to the duration of UV irradiation. The control film exhibits a higher integral area ratio than the BTTM perovskite film. Moreover, the area ratio of the BTTM film remains relatively stable after aging, indicating that BTTM effectively mitigates UV-induced degradation of the perovskite structure. Additionally, UV–visible spectroscopy measurements were performed on both the control group and the BTTM perovskite films, and tauc plots were fitted (Fig. S13), and the introduction of BTTM did not alter the optical bandgap of the perovskite film. Moreover, the BTTM perovskite film exhibits better retention of absorbance after UV irradiation, further demonstrating the UV-protective effect of BTTM. Subsequently, SEM images were used to observe morphological changes in both control and BTTM perovskite films after exposure to 365-nm UV light (Fig. 3H). Interestingly, the SEM images of the films became blurred after UV irradiation, potentially related to changes in surface conductivity. Simultaneously, noticeable gaps appeared at grain boundaries, indicating that UV irradiation primarily affects grain boundaries, subsequently triggering perovskite degradation.

**Fig. 4.**
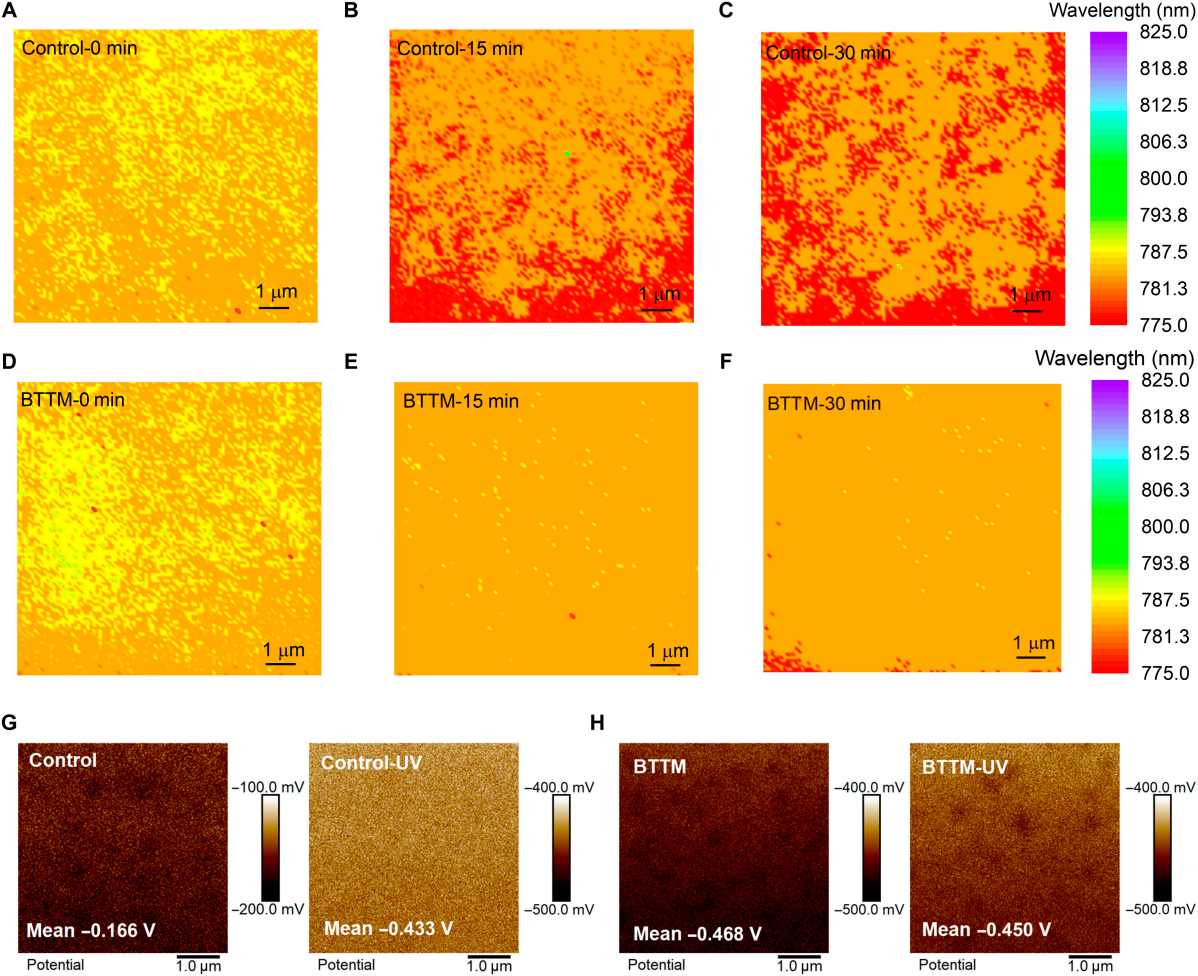
Comparison of films uniformity under the ultraviolet (UV) irradiation between control and 2,3-bis(2,4,5-trimethyl-3-thienyl) maleimide (BTTM) perovskite films. (A to C) Photoluminescence mapping images of control perovskite films before and after UV irradiation. (D to F) Photoluminescence mapping images of BTTM perovskite films before and after UV irradiation. (G) Kelvin probe force microscopy (KPFM) images of control perovskite films before and after UV irradiation. (H) KPFM images of BTTM perovskite films before and after UV irradiation.

The heterogeneity of halide perovskites severely limits the development of device optoelectronic performance and stability, particularly during UV aging processes. Therefore, we further investigated the effect of BTTM on the homogeneity of perovskite films after UV aging. Here, both control and BTTM perovskite films were exposed to 365-nm UV light at an irradiance of 200 mW/cm^2^, and their PL mapping images were measured as shown in Fig. 4A to F and Figs. S14 and S15. The results reveal that the BTTM film exhibits enhanced homogeneity after UV irradiation, whereas the control perovskite film shows deteriorated PL peak position uniformity and a tendency toward blueshift, consistent with the findings in Fig. 3B. Additionally, Fig. S14 shows an increase in the PL intensity of the perovskite film after UV irradiation, which may be attributed to the high energy provided by UV irradiation compensating for defects. Furthermore, Fig. S15 indicates that the half-width of the perovskite film’s peak position broadens after UV irradiation. To further elucidate the effect of UV irradiation on perovskite film uniformity, Kelvin probe force microscopy measurements were performed on both control and BTTM films before and after UV irradiation, as shown in Fig. 4G and H, with a cumulative irradiation dose of 2 kWh/m^2^. It can be observed that the surface potential of the control perovskite film decreased significantly from −0.166 to −0.433 V after UV irradiation, while the surface potential of the BTTM perovskite film showed no marked change, increasing only slightly from −0.468 to −0.450 V. This indicates that after UV irradiation, the anchoring of BTTM to the components within the perovskite film effectively maintains the uniformity.

**Fig. 5.**
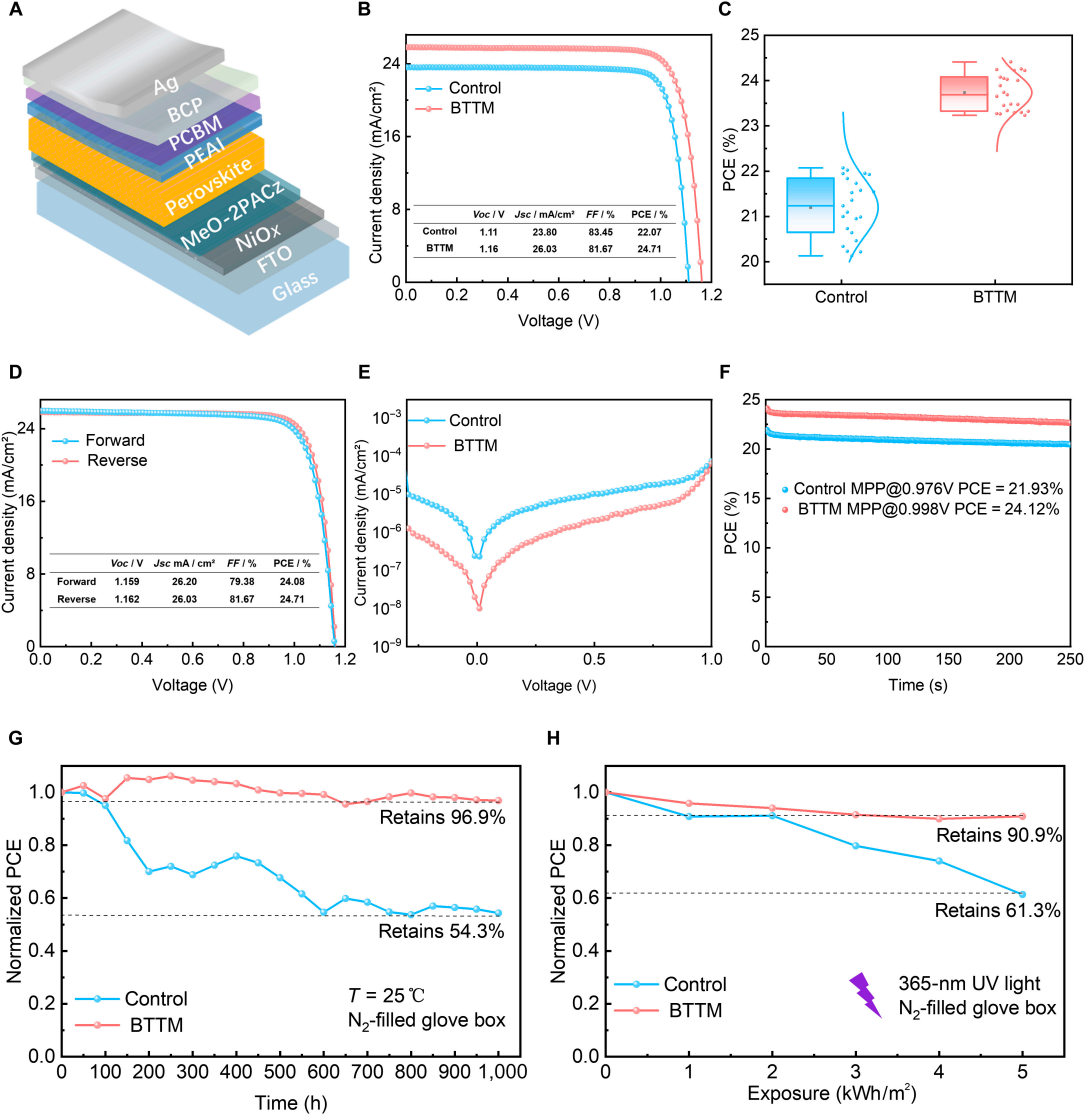
Performance and stability of control and 2,3-bis(2,4,5-trimethyl-3-thienyl) maleimide (BTTM) devices. (A) Schematic diagram of device structure. (B) *J*-*V* curves of control and BTTM devices. (C) Control and BTTM devices’ power conversion efficiency (PCE) distribution box plots. (D) Forward and reverse scan *J*-*V* curves of BTTM devices. (E) Dark *J*-*V* curves of control and BTTM devices. (F) Steady-state power output of control and BTTM devices. (G) Nitrogen atmosphere stability of perovskite solar cells (unencapsulated). (H) PCE stability under ultraviolet (UV) irradiation (365 nm).

PSCs with the structure of F-doped tin oxide (FTO)/NiO*_x_*/MeO-2PACz/perovskite/2-phenylethylamine hydroiodide (PEAI)/(6,6)-phenyl-C61-butyric acid methyl ester (PCBM)/bathocuproine (BCP)/Ag were fabricated, as shown in Fig. 5A, to investigate the effect of BTTM on PSC performance. Figure 5B displays the *J*-*V* curves of the optimal control and BTTM devices, where the optimal BTTM PSCs exhibit a *Voc* of 1.16 V, a short-circuit current density (*Jsc*) of 26.03 mA/cm^2^, a fill factor (*FF*) of 81.67%, and a PCE of 24.71%, while the control device shows a lower PCE of 22.07%. Box plots of PCE and *J*-*V* parameter distributions for control and BTTM devices are shown in Fig. 5C and Fig. S16, demonstrating improved performance parameters across all devices. Additionally, forward and reverse scan tests were conducted on a BTTM device, as shown in Fig. 5D, revealing minimal hysteresis between forward and reverse scans. In order to explore the applicability of this method in other perovskite compositions, we prepared wide-bandgap PSCs. The best PCE of the BTTM device increased from 22.02% in the reference device to 23.50% (Fig. S17). To understand the impact of BTTM adding on charge recombination dynamics in the devices, we measured dark current curves for both control and BTTM devices (Fig. 5E). Compared to the control device, the BTTM device shows a lower saturation current density, indicating reduced charge recombination and thus higher *Voc*. Figure 5F displays the steady-state power output of PSCs biased at the maximum power point voltage for both control and BTTM devices, showing a stabilized PCE of 24.12% for BTTM devices. When unencapsulated PSCs were stored in a nitrogen environment for 1,000 h, the PCE changes are shown in Fig. 5G. The BTTM device maintained 96.9% of its initial PCE after 1,000 h, while the control device retained only 54.3%, indicating better long-term stability for BTTM devices. To evaluate the protective effect of BTTM against UV irradiation in PSCs, we subjected both control and BTTM devices to UV irradiation in a N_2_ glovebox. During the test, it was found that the NiO*_x_* layer and the PCBM layer have a notable impact on the UV stability of the device [56,57]. Under UV irradiation, the PCE of the device drops rapidly, as shown in Figs. S18 and S19. Therefore, we chose FTO/MeO-2PACz/perovskite/PEAI/C_60_/BCP/Ag device structure for UV stability test, as shown in Fig. 5H, with a total UV dose of 5 kWh/m^2^. Throughout the UV aging test, the control device’s PCE gradually degraded to only 60% of its initial value, while the BTTM device maintained 90% of its initial PCE, demonstrating the significant role of BTTM in enhancing the UV stability of perovskite devices.

## Conclusion

In this study, we incorporated the photoisomeric BTTM molecule capable of interconverting between UV and visible light into perovskite. This enhanced the photovoltaic performance of PSCs while simultaneously improving their UV stability. The incorporation of BTTM promotes perovskite crystal growth and passivates defects within the film, significantly increasing the *Voc* of PSCs. This results in a PCE improvement from 22.07% to 24.71%. Under UV irradiation, BTTM mitigate perovskite degradation by suppressing halide ion migration. After cumulative exposure to 5 kWh/m^2^ of continuous UV radiation, BTTM PSCs retained 90% of initial PCE, whereas unmodified PSCs exhibited gradual degradation to only 60% of their initial PCE, demonstrating markedly enhanced UV stability. This study provides deeper insights into perovskite aging under UV irradiation and offers a simple additive engineering approach for UV protection in halide perovskites, presenting novel strategies for PSCs in environments with intense UV exposure.

## Materials and Methods

Comprehensive materials and methods can be found in the Supplementary Materials.

## Data Availability

All data required to evaluate the paper’s conclusions are available in the paper or the Supplementary Materials.
