# Supplementary material for: Photoisomeric Molecule-Mediated Ion Anchoring and UV Resistance in Metal Halide Perovskites
Source: Research (Wash D C). 2026 Mar 18;9:1196. doi: 10.34133/research.1196 (PMC12996635; doi:10.34133/research.1196)
Supplement: Supplementary 1 — Materials and Methods Figs. S1 to S19 Tables S1 to S4 [file research.1196.f1.docx]

Supplementary Materials

**Photoisomeric Molecule-Mediated Ion Anchoring and UV Resistance in Metal Halide Perovskites**

Wenying Zhao^1†^, Duo Qu^1†^, Yongli Zhang^2,3†^, Chuanzhen Shang^1^, Xuewen Zhang^1^, Chenyun Wang^1^, Bin Zhou^1^, Jingyuan Qiao^1^, Ruilin Han^1^, Shasha Wang^1^, Yuyao Bi^1^, Siyu Wei^1^, Zheng Bao^4^, Fan Xu^4,5^, Fengjun Ye^4^, Yongguang Tu^1^*

^1^ State Key Laboratory of Flexible Electronics (LOFE) & Institute of Flexible Electronics (IFE), Shaanxi Key Laboratory of Flexible Electronics, MIIT Key Laboratory of Flexible Electronics (KLOFE), Northwestern Polytechnical University, Xi'an, 710072, China.

^2^ Harbin Institute of Technology, No. 92 Xidazhi Street, Nangang District, Harbin City, Heilongjiang Province, 150001, China.

^3^ Shanghai Institute of Space Power-Sources, No. 2965 Dongchuan Road, Minhang District, Shanghai 200245, China.

^4^ Beijing Solarverse Optoelectronic Technology Co., Ltd, Beijing, 100176, China.

^5^ Shenzhen Institute for Advanced Study, University of Electronic Science and Technology of China, Shenzhen 518110, China.

^†^ These authors contributed equally to this work.

^*^ Address correspondence to: Yongguang Tu; iamygtu@nwpu.edu.cn

**Experimental section**

**Materials**

Patterned ITO glass is provided by Shenzhen South China Xiang Cheng Technology Co. Patterned FTO glass is provided by Liaoning You xuan New Energy Technology Co. The solvents, including isopropanol (IPA, 99.8% Extra Dry), N, N-dimethylformamide (DMF, 99.8%Extra Dry), dimethyl sulfoxide (DMSO, 99.7%+ Extra Dry), chlorobenzene (CB, ≥99.8% Extra Dry), and ethanol (EtOH, 99.5% Extra Dry), were all purchased from Acros Organics. Nickel oxide (NiOx,99%) was purchased from Liaoning You xuan New Energy Technology Co. High-purity MeO-2PACz, PbI_2_ (99.999%) andPbBr_2_(99.999%) are provided by Tokyo Chemical Industry Co, Ltd (TCI). FAI was acquired from Greatcell Solar Co., Ltd (Dyesol). Additional materials including Cesium iodide (CsI), methylammonium bromide (MABr), methylammonium chloride (MACl), phenethyl ammonium iodide (PEAI), PCBM, C_60_ and BCP were all purchased from Xi’an Polymer Light Technology Corp. In addition, 2,4, 5-trimethyl-3-butylmaleimide (BTTM) was purchased from Maclean Corporation.

**Precursor solution**

To prepare the perovskite precursor solution, this work meticulously measured out PbI_2_ (572.0 mg), FAI (196.1 mg), PbBr_2_ (22.0 mg), CsI (18.2 mg), MACl (8.8 mg), and MABr (6.7 mg). Subsequently, a mixed solvent system of DMF (800μL) and DMSO (200μL) was added to the reaction system. Under magnetic stirring conditions, slowly heat the solution to 60℃ to ensure that all components are fully dissolved and reach a uniformly mixed state. The perovskite precursor solution of the control group was not added with BTTM. The addition amount of the perovskite precursor solution of the BTTM experimental group was calculated according to the corresponding mass concentration percentage.

**Device Fabrication**

Before use, the substrate must be strictly cleaned. The patterned ITO glass substrates were successively cleaned with pure water, acetone, detergent and isopropyl alcohol. Each solvent was cleaned in the ultrasonic cleaning tank for 15 minutes. Subsequently, the cleaned ITO substrates were placed in an oven and dried at 60℃ for 5 hours. To further enhance the quality of the substrate, the dried ITO glass was treated with ultraviolet ozone for 20 minutes before being set aside. This step can effectively remove the residual oxygen-containing functional groups on the surface. Subsequently, the processed substrates are transferred to the air glove box for hole transport layer (HTL) deposition. The prepared NiOx solution (10 mg/ml in H_2_O) was spin-coated onto the substrate at 4000 rpm for 30 seconds in an air glove box, and then annealed at 100°C for 10 minutes. After annealing, the substrate is transferred to a nitrogen glove box for subsequent spin coating. The MeO-2PACz was spin-coated at 3000 rpm for 30 seconds and then annealed at 100°C for 10 minutes. The perovskite precursor solution was spin-coated on the MeO-2PACZ layer, first at 200 rpm·s^-1^ to 1000 rpm for 5 s, then ramping up to 3000 rpm·s^-1^ to 4000 rpm for 30 s. After the spin coating is completed, the films were placed in a 100℃ heating box and continuously heated for 60 minutes. The prepared perovskite films need to undergo subsequent post-treatment. First, the PEAI solution (1.5mg/ml in IPA) was spin-coated at 3000 rpm for 30 seconds and then annealed at 100°C for 10 minutes. This process can ensure the best quality and good stability of the film. As the electron transport layer (ETL), PCBM solution (20mg/ml in CB) was spin-coated at 1500 rpm for 30 seconds and then annealed on a hot table at 100 ° C for 5 minutes. Finally, through a precise mask, three layers of C_60_ (15nm), BCP (7nm), and Ag(100nm), were successively deposited under vacuum conditions.

**Characterization**

UV-vis optical absorption spectra were measured using a spectrophotometer (UH4150, Hitachi, Japan). Fourier-transform infrared (FTIR) was collected using a Bruker Tensor II with an ATR accessory (Bruker, Tensor II, Germany). X-ray photoelectron spectroscopy (XPS) spectra were obtained from a Thermo Fisher Scientific ESCALAB 250XI under ultrahigh vacuum conditions with a monochromatic Al Kα (1486.68 eV) X-ray source. The XPS spectra were calibrated using the binding energy of C1s at 284.6 eV. X-ray diffraction (XRD) data were collected using the D8 Advance instrument (Bruker) equipped with Cu Kα radiation (λ=0.01542 nm, 40 kV, 40 mA). Scanning electron microscope (SEM) images were obtained using the Gemini SEM 300 instrument (Zeiss, Germany). Steady-state photoluminescence (PL) was excited by Xenon lamp and recorded using an Edinburgh FLS 1000 spectrometer. The time-resolved photoluminescence (TRPL) spectra were also collected using the Edinburgh FLS 1000 spectrometer. Grazing-incidence X-ray diffraction (GIXRD) test was conducted using the nano ix-we system (Rigaku Co., Tokyo, Japan), with various ψ angles (0°–50°). PL mapping images were excited by a 780 nm laser with a power of 10μW using a laser scanning confocal microscope (Enlitech, SPCM-1000). Atomic force microscopy (AFM) images were captured by the Bruker Dimension Icon instrument (Bruker, Germany). Absolute photoluminescence quantum yield (PLQY) was recorded using a Quanta-Phi integrating sphere with a Fluorolog system under the excitation wavelength of 450 nm.

**Device measurement**

The current density-voltage (J-V) curves of the solar cells were precisely measured using a Keithley 2400 Series Source Meter, powered by a 150 W AAA solar simulator

(CME-Sol 8050, Microenerg (Beijing) Technology Co., Ltd.). The measurements were conducted under simulated AM 1.5 G sunlight, maintaining an irradiance of 100 MW·cm^-2^. The active area of solar cell was precisely limited to 0.062 cm^2^ using a custom-made mask. All measurements were carried out in an unencapsulated natural environment. Prior to testing, light intensity calibration was carried out using standard mono-crystalline silicon solar cells equipped with a KG-5 filter to ensure measurement accuracy. The J-V measurements were executed in both reverse (1.22 V → -0.02 V, scan rate 40 mV/s) and forward (-0.02 V → 1.22 V, scan rate 40 mV/s) scans, without any delay time. In addition, the stable power output (SPO) and ultraviolet stability were also evaluated under the same simulated solar radiation conditions. The ultraviolet stability of the devices were tested by irradiation with a 365nm ultraviolet lamp, and the test intensity was marked by a radiometer.

**Figures**


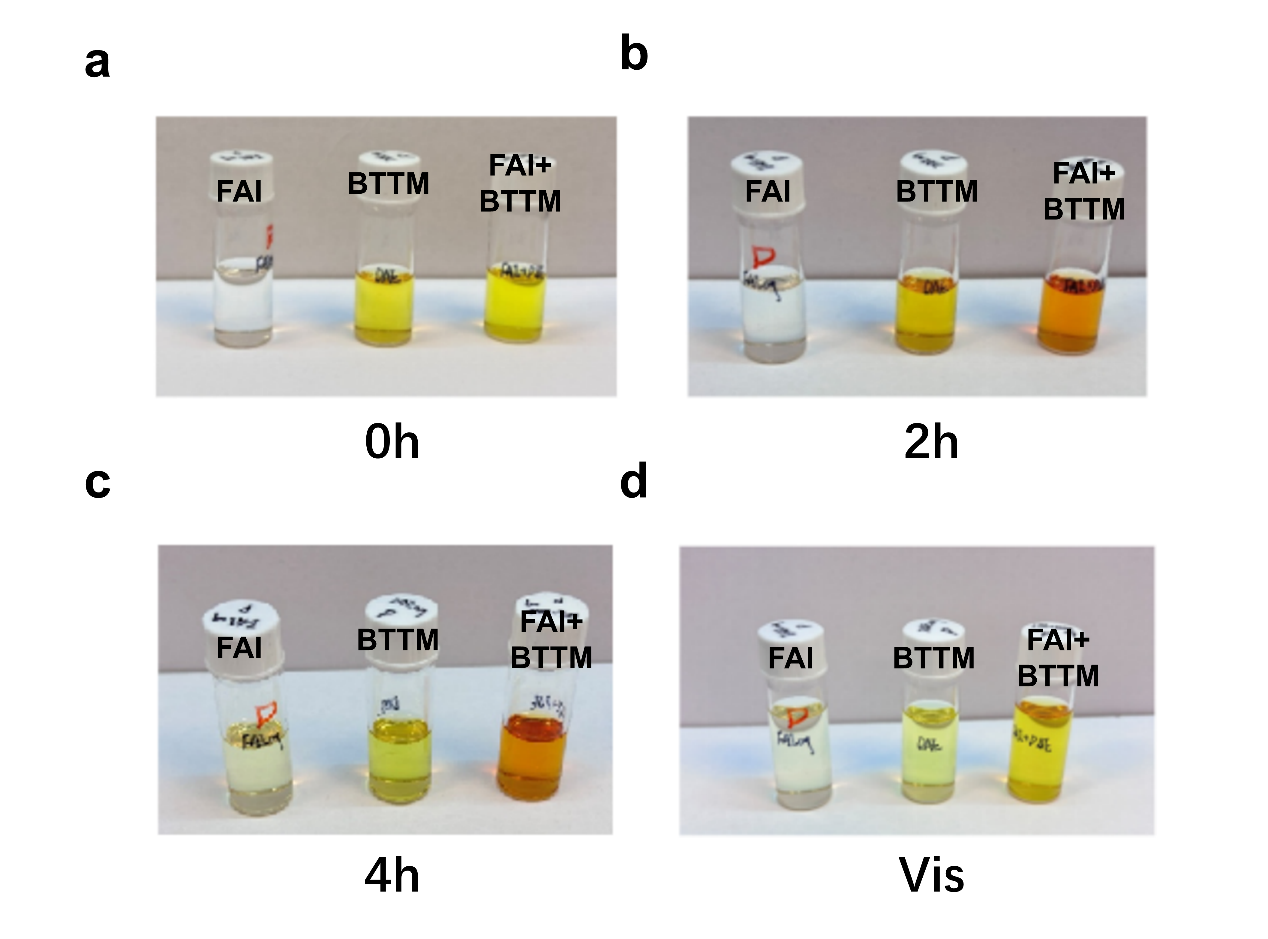


**Fig. S1.** The color changes of FAI, BTTM, and FAI+BTTM solutions dissolved separately in IPA under different durations of UV light (365nm) irradiation. The samples a) without UV irradiation, b) after 2 hours of UV irradiation, c) after 4 hours of UV irradiation. d) The solution that has been allowed to recover for a period of time after being irradiated with UV irradiation.


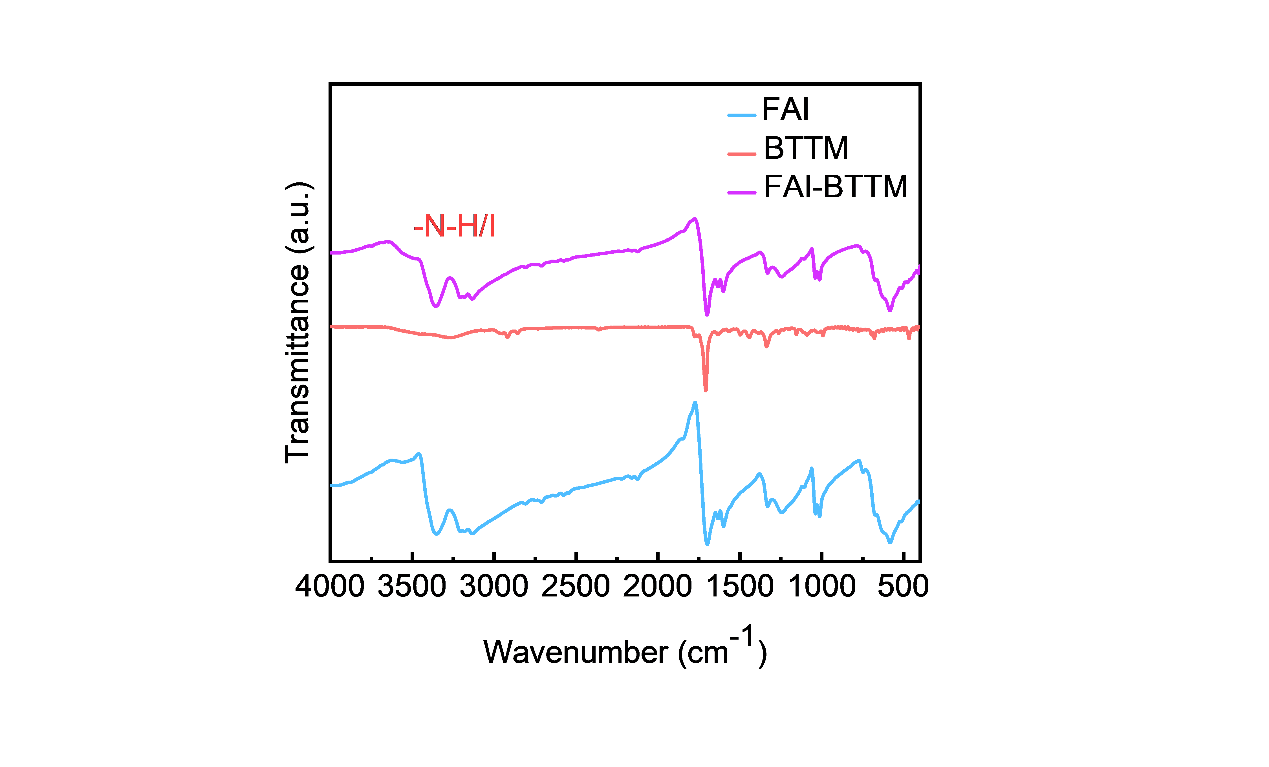


**Fig. S2.** FTIR spectra of the interaction between FAI and BTTM.


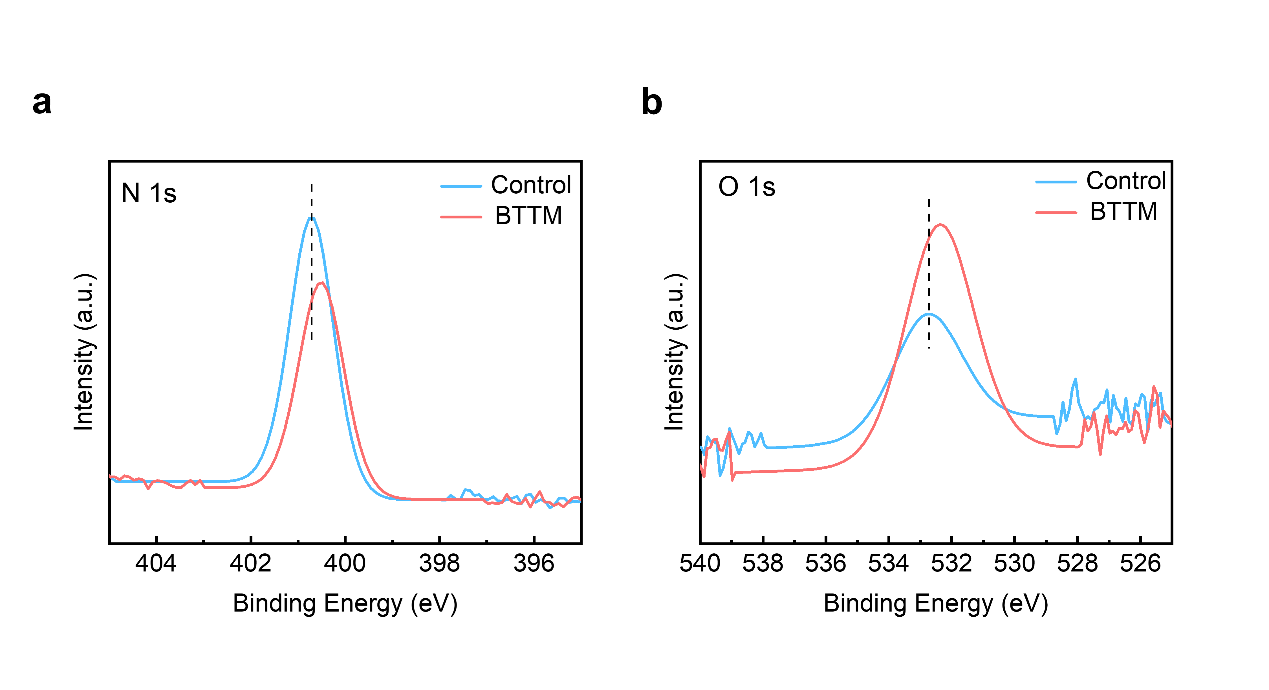


**Fig. S3.** The XPS spectrums of Control and BTTM perovskite films. a) The N 1s XPS spectrums. The N 1s peak position of BTTM perovskite film shows a significant shift compared to that of the Control perovskite film. b) The O 1s XPS spectrums. The O 1s peak position of BTTM perovskite film shows a significant shift to lower energy levels compared to the Control perovskite film.


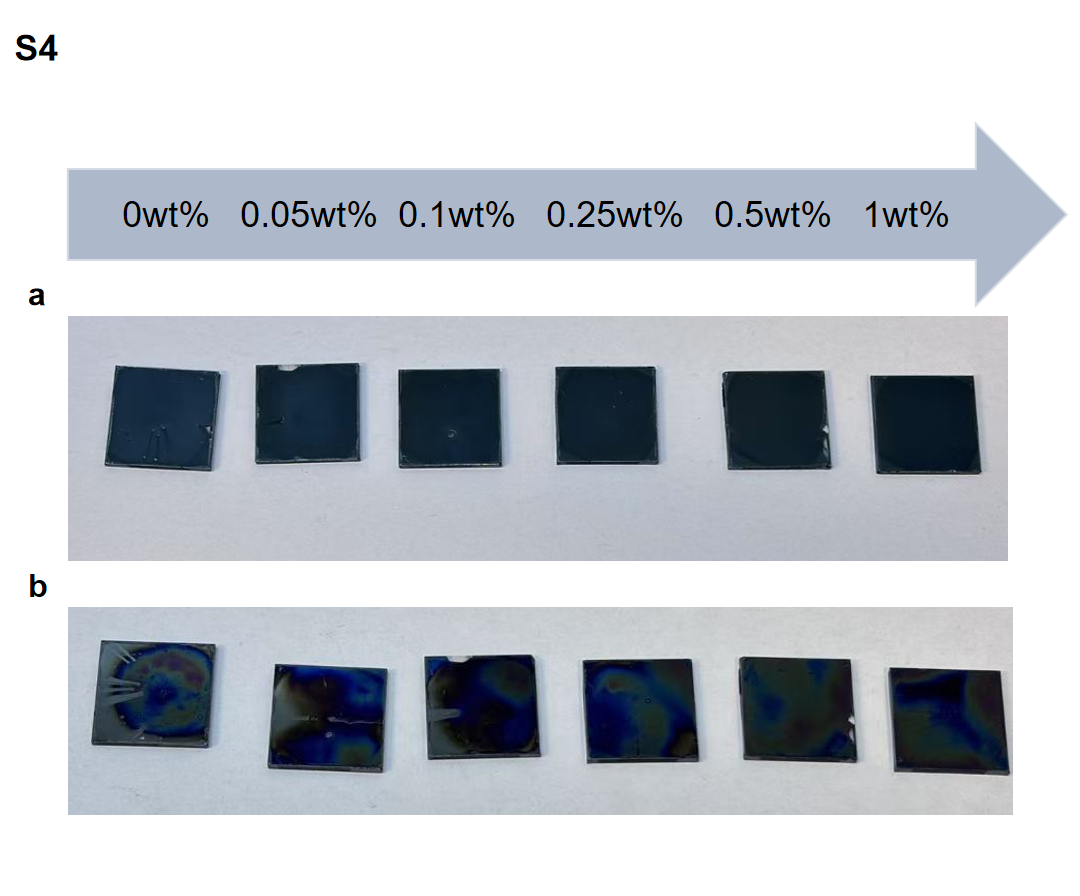


**Fig. S4.** Photographs of Perovskite films with different BTTM contents. a) Without UV light. b) After UV irradiation. The 0.1wt% BTTM perovskite film maintains its morphology well after UV irradiation.


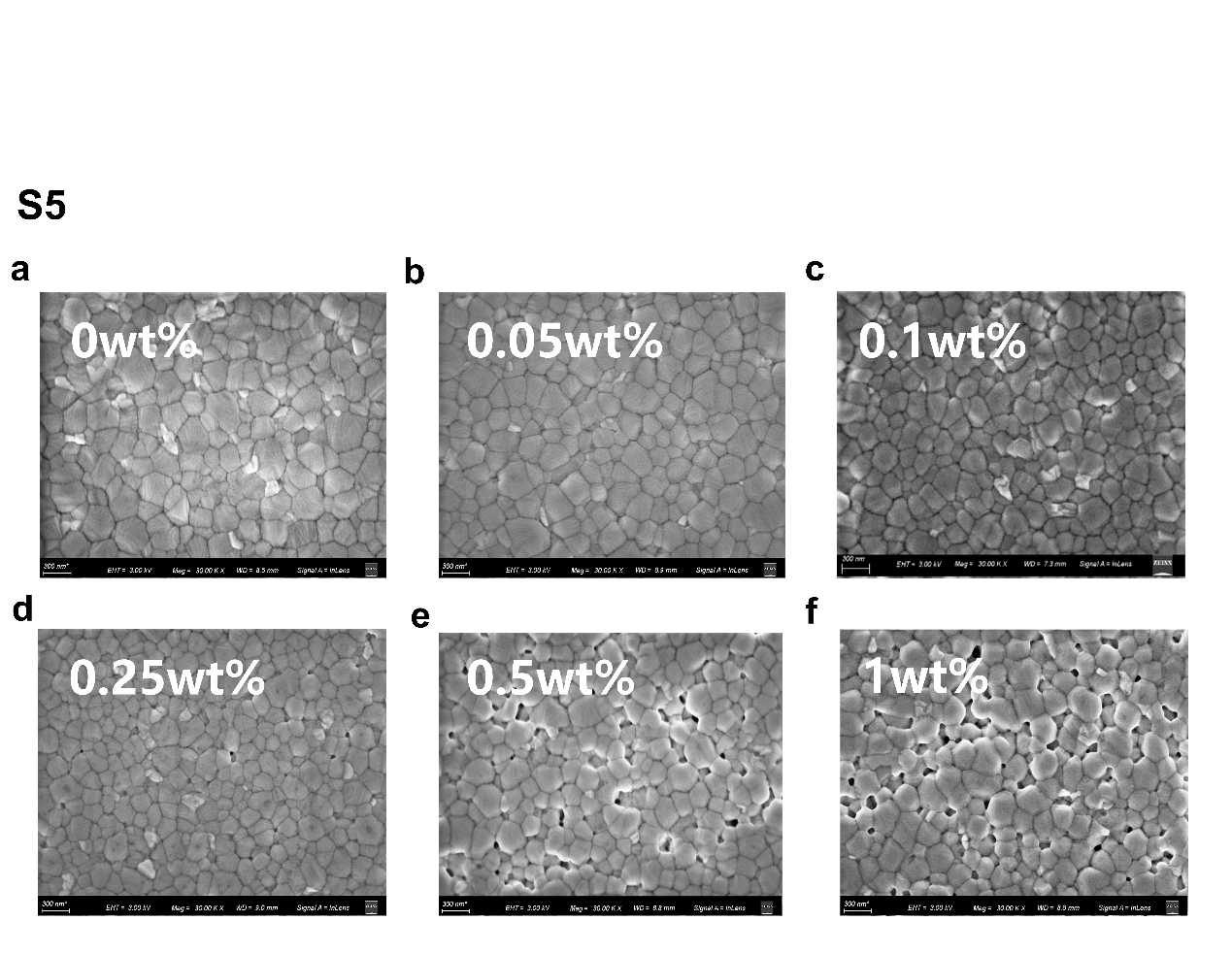


**Fig. S5.** The SEM morphology of gradient concentration of BTTM perovskite films. The perovskite films without BTTM show smaller grains and a large amount of PbI_2_. With the addition of 0.05wt% and 0.1wt% BTTM, the grain size of perovskite films increases and the PbI_2_ content decreases. However, when the BTTM content is too high, the morphology of perovskite films deteriorates, resulting in many voids. Therefore, the optimal BTTM content is determined to be 0.1wt%.


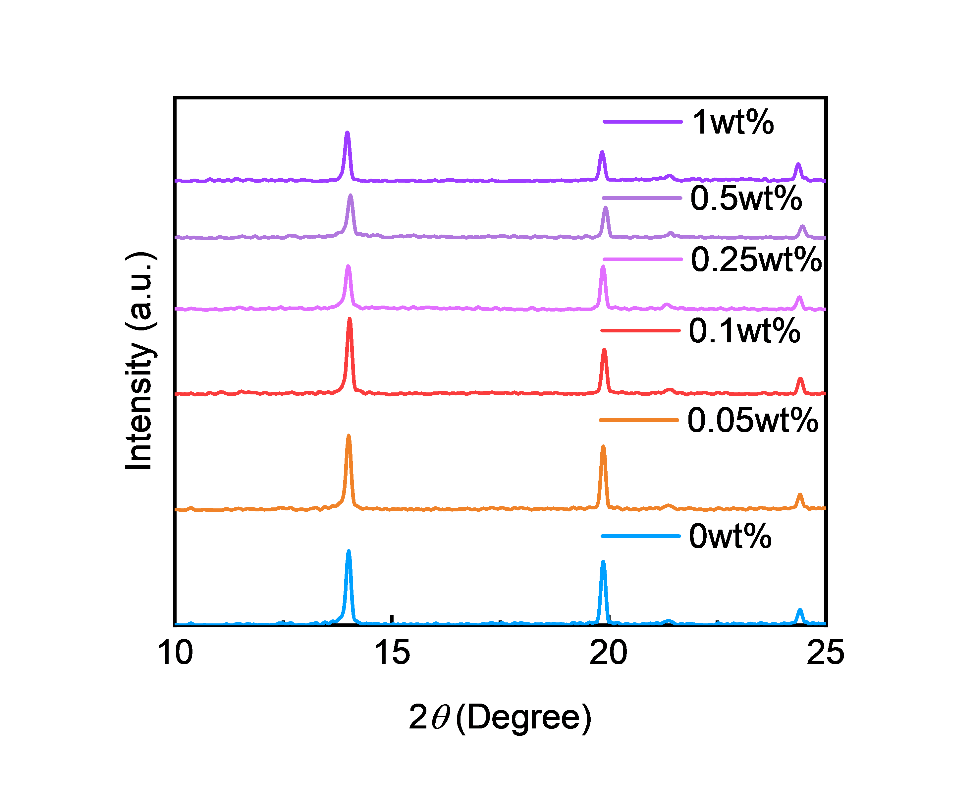


**Fig. S6.** The XRD patterns of gradient concentration of BTTM perovskite films. After adding BTTM, the relative intensity of the (001) crystal plane increased, indicating that BTTM can regulate the growth of perovskite crystals. However, excessive addition of BTTM will lead to a decrease in peak intensity.


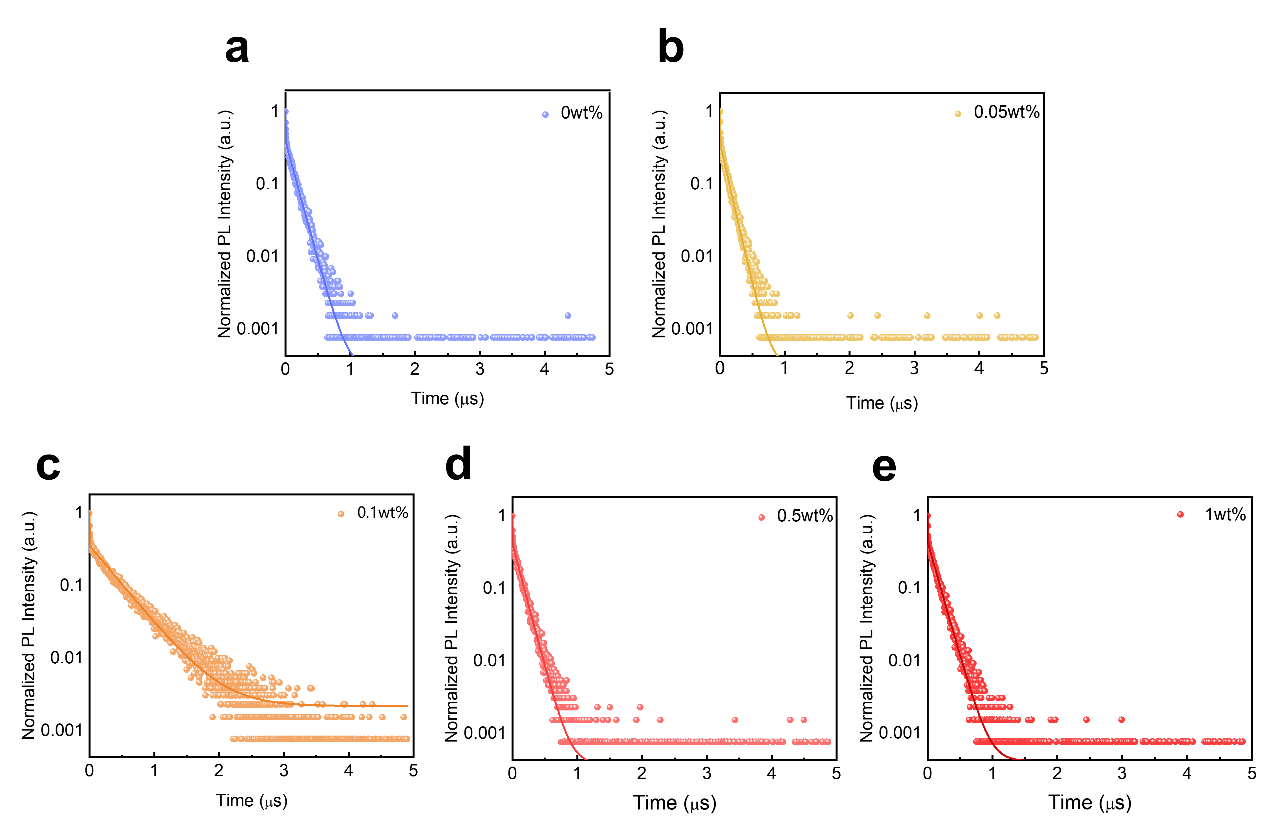


**Fig. S7.** TRPL spectra of perovskite films with different BTTM contents. By comparison, it is found that the perovskite thin film with 0.1wt% BTTM addition has the longest carrier lifetime.


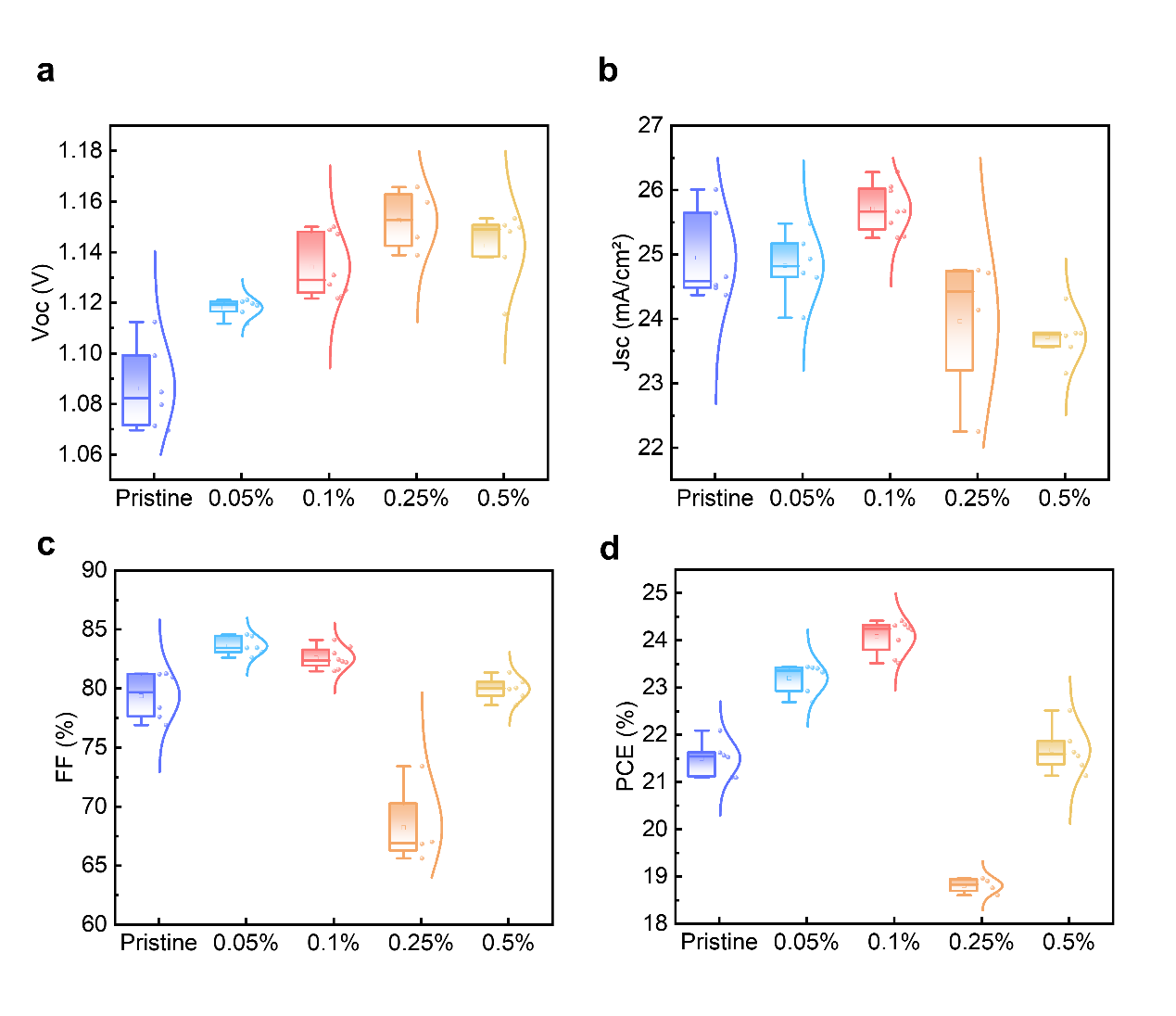


**Fig. S8.** The J-V parameters statistical distribution of different BTTM contents devices.

a) V_OC_, b) J_SC_, c) FF, and d) PCE.


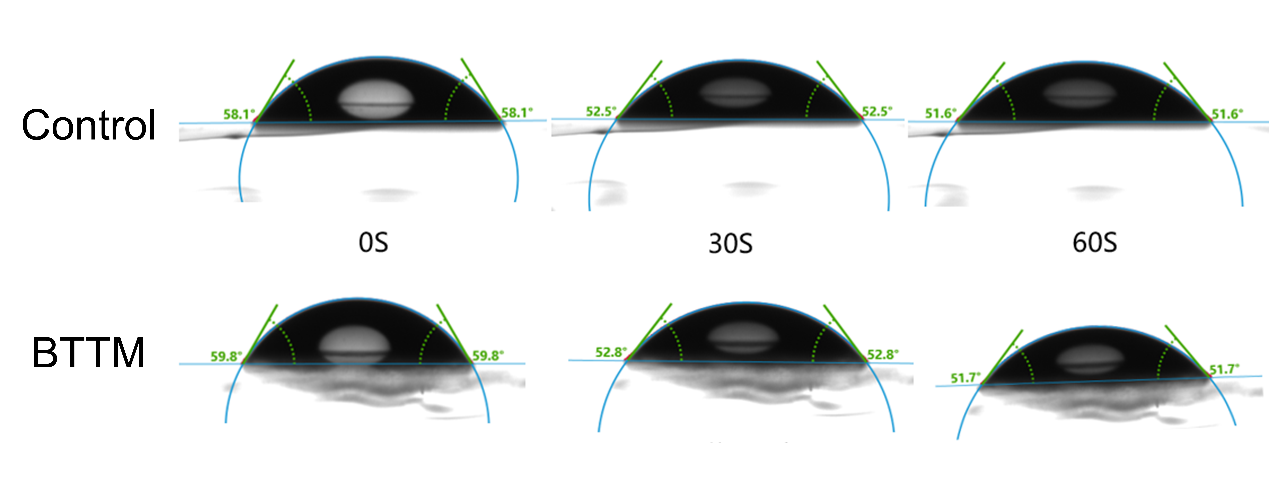


**Fig. S9.** Contact angle measurement of Control and BTTM perovskite films. During the test, water droplets were placed on the perovskite films, and the contact angle was observed at 0s/30s/60s.

**
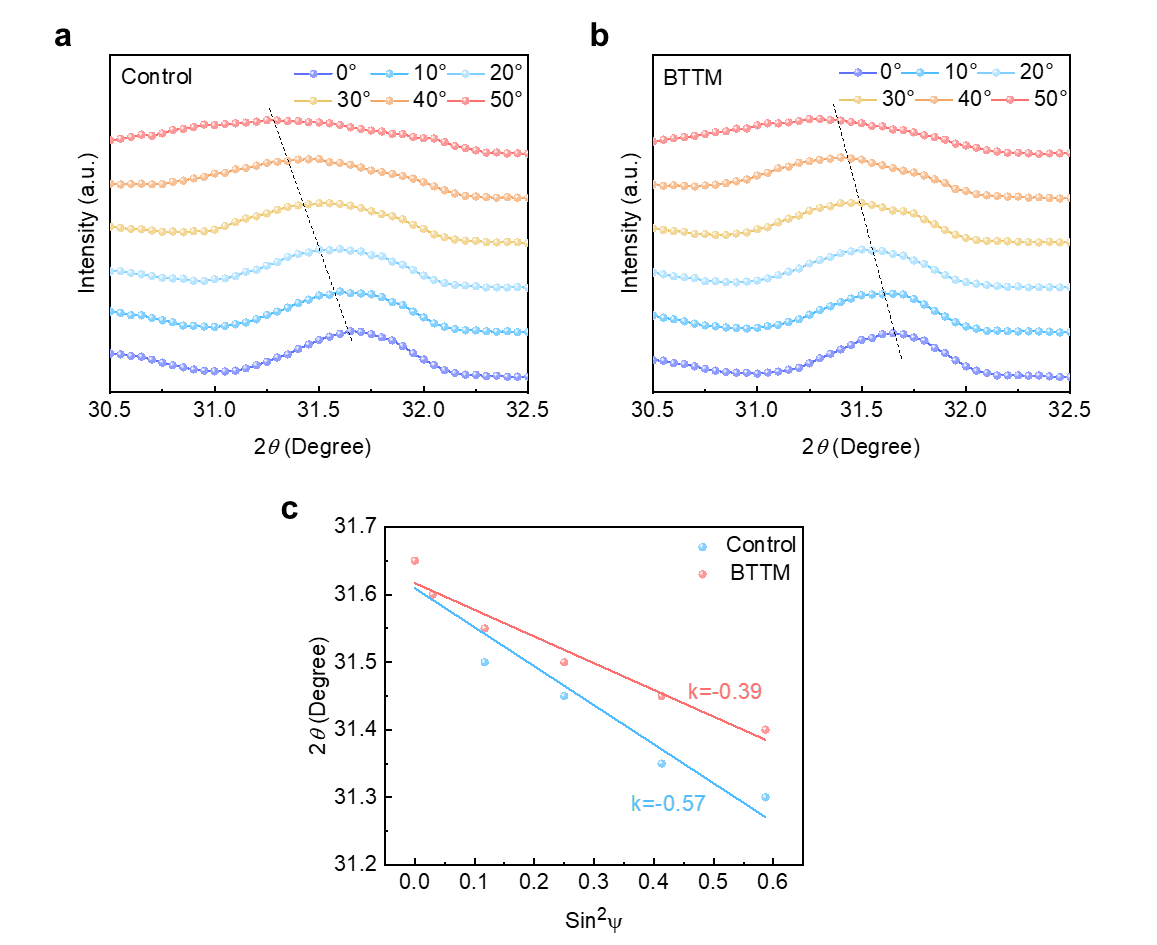
**

**Fig. S10.**GIXRD tests of the Control and BTTM perovskite films.

a-b) GIXRD patterns of the Control and BTTM perovskite films. c) Comparison chart of GIXRD tangent slope between the Control and BTTM perovskite films.


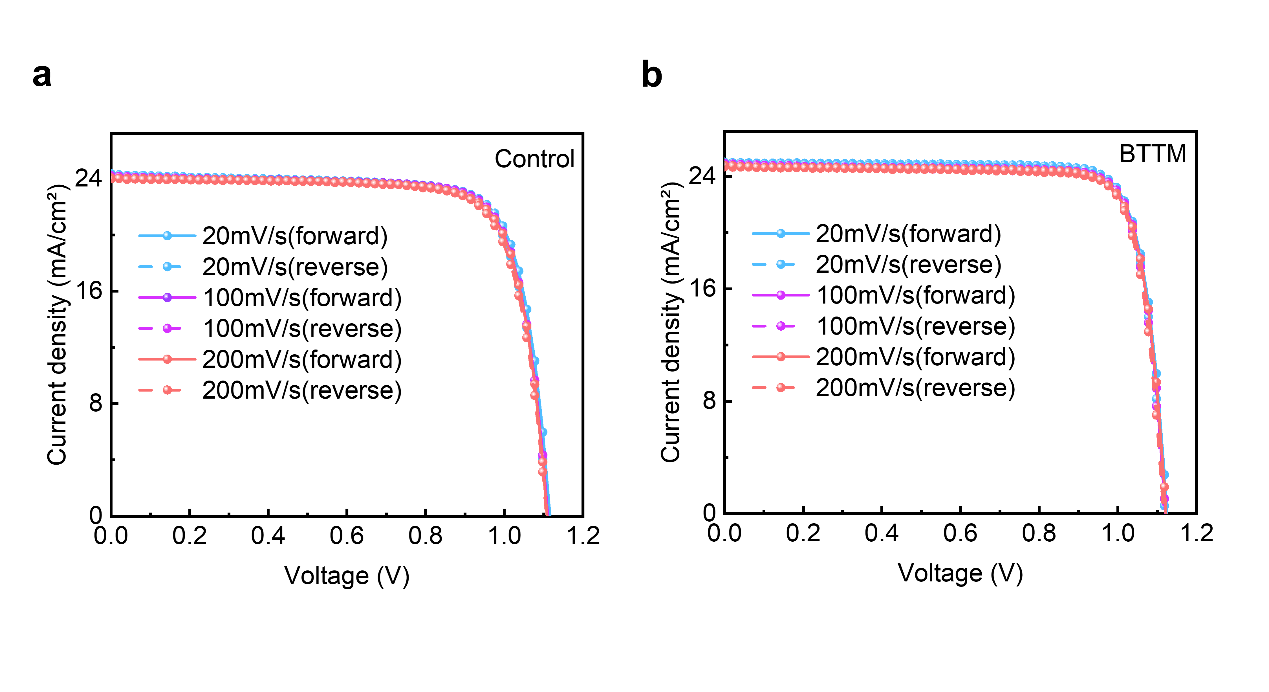


**Fig. S11.** J-V curves of Control(a) and BTTM(b) devices at different scan-rates.


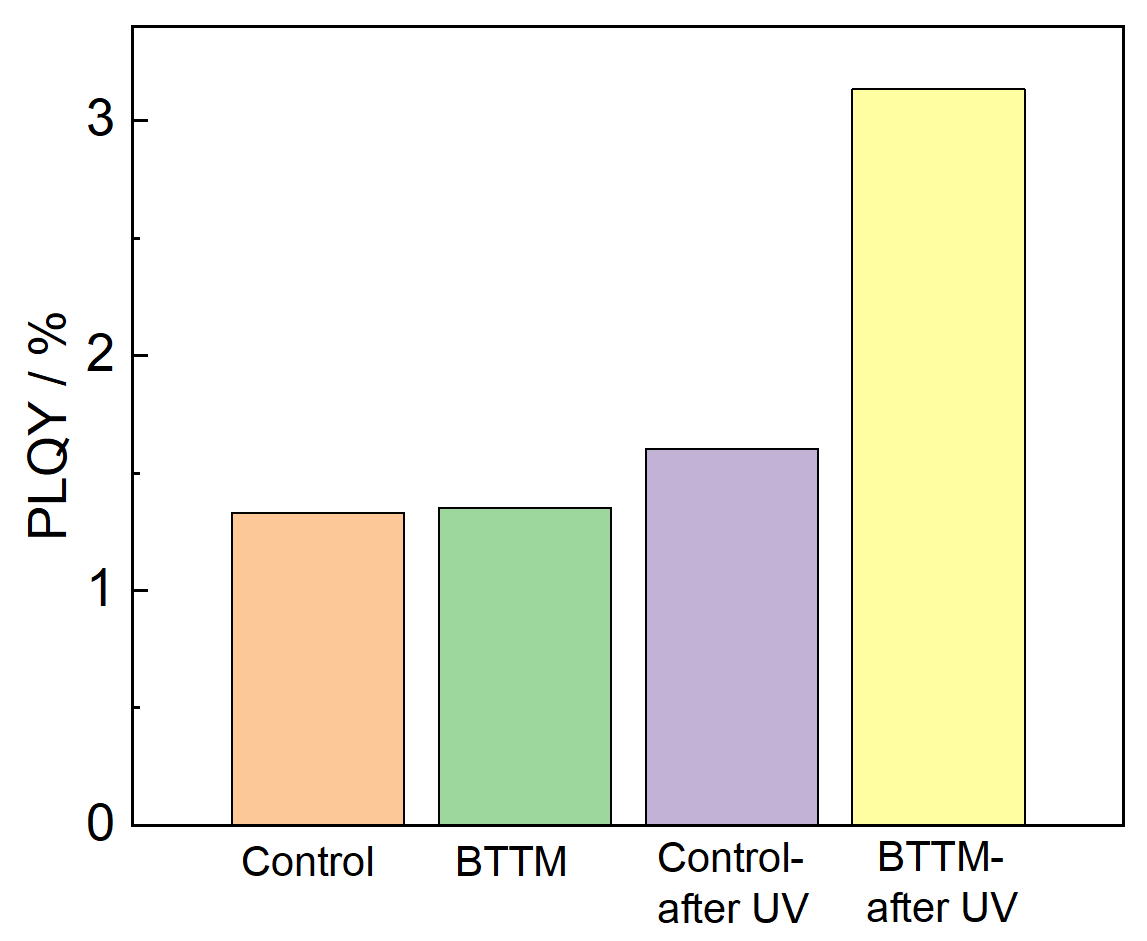


**Fig. S12.** Comparison of the photoluminescence quantum yield (PLQY) between the reference and BTTM perovskite films before and after UV aging.

**
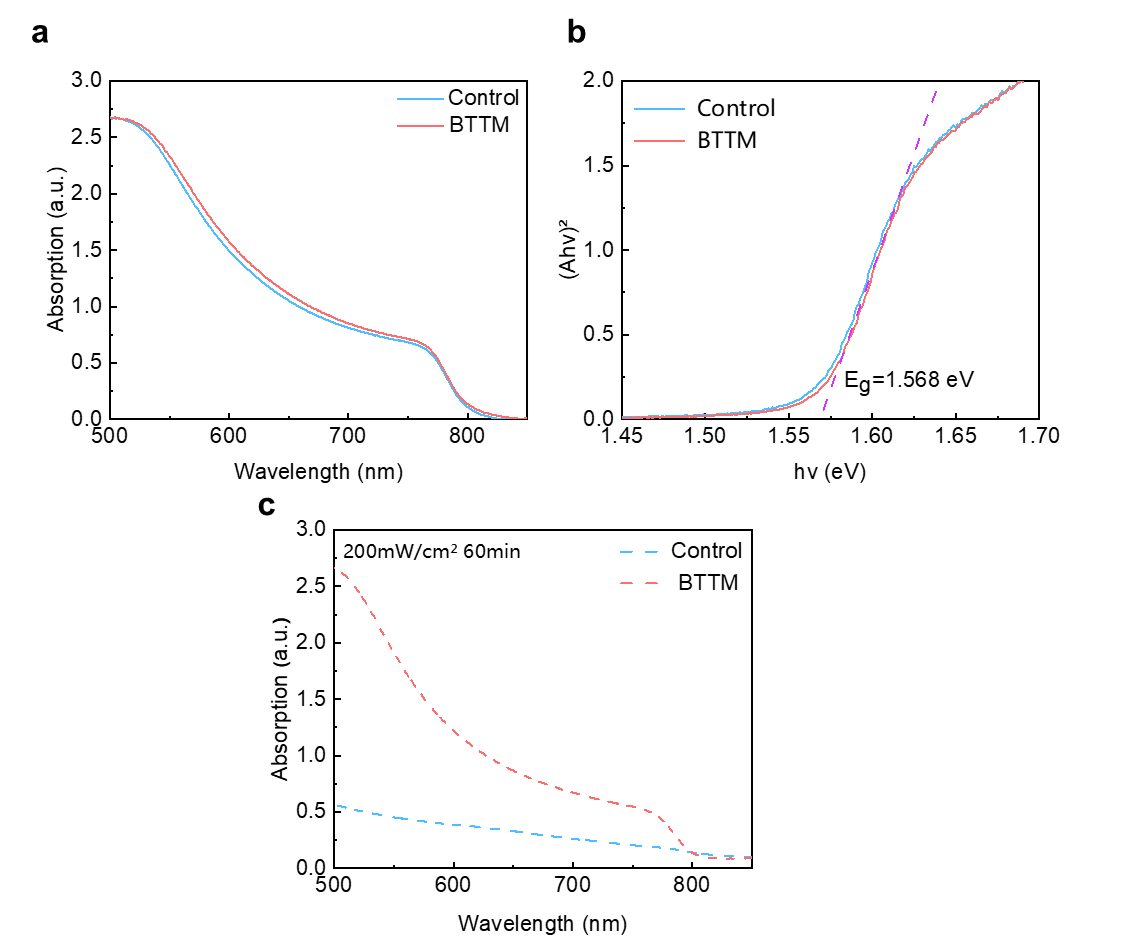
**

**Fig. S13.** Absorption spectra of Control and BTTM perovskite films before a) and after c) UV irradiation. b) Tauc plots of the control and BTTM perovskite films.


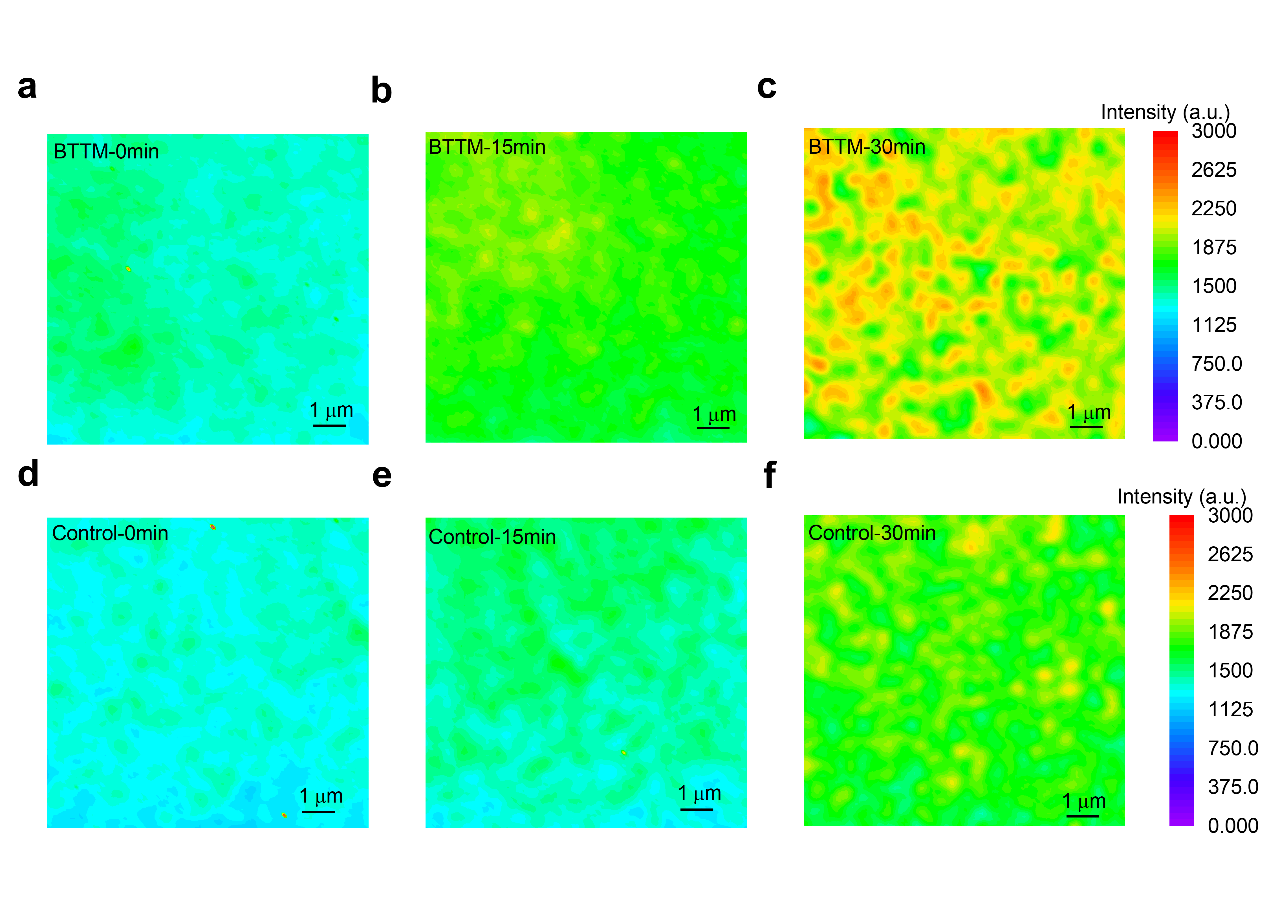


**Fig. S14.** PL mapping images of Control and BTTM perovskite films under UV irradiation (peak intensity).


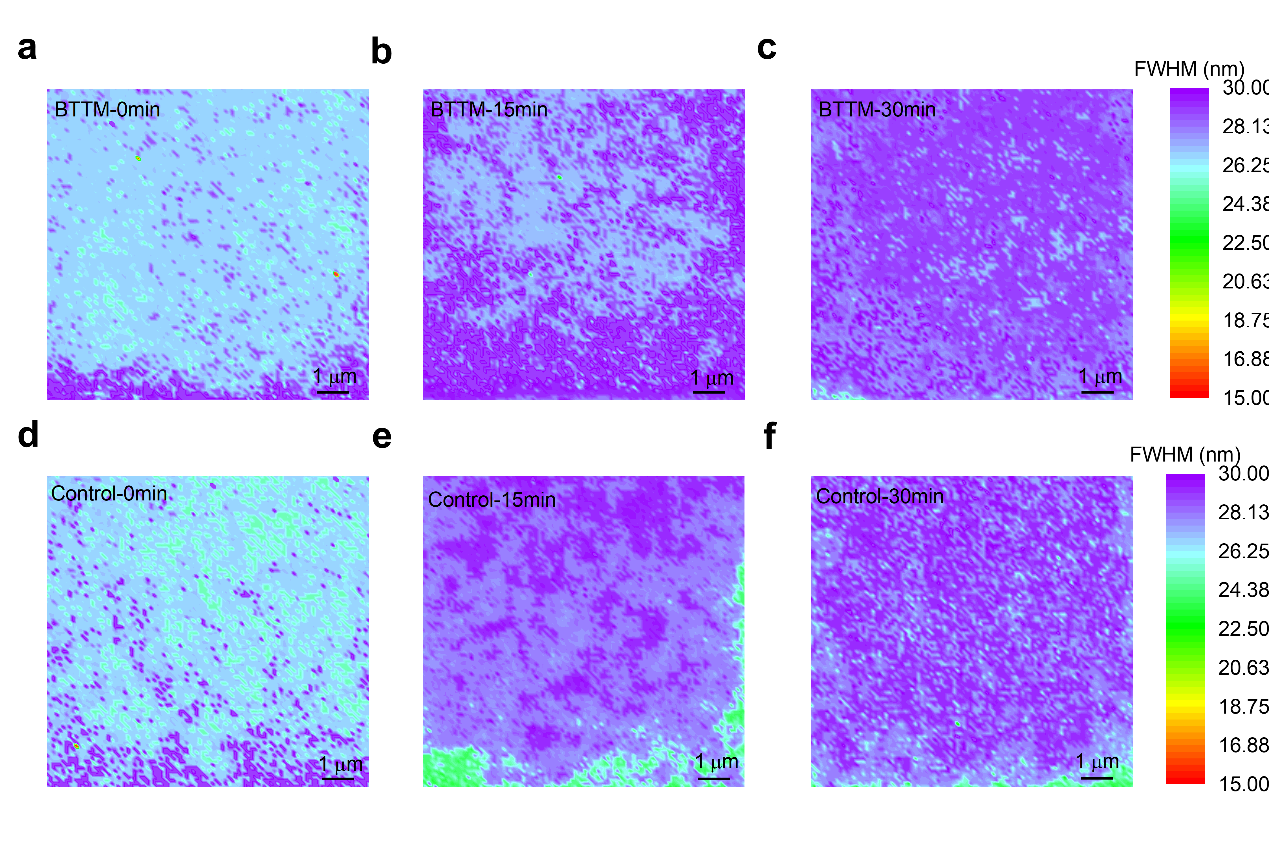


**Fig. S15.** PL mapping images of Control and BTTM perovskite films under UV irradiation (FWHM).


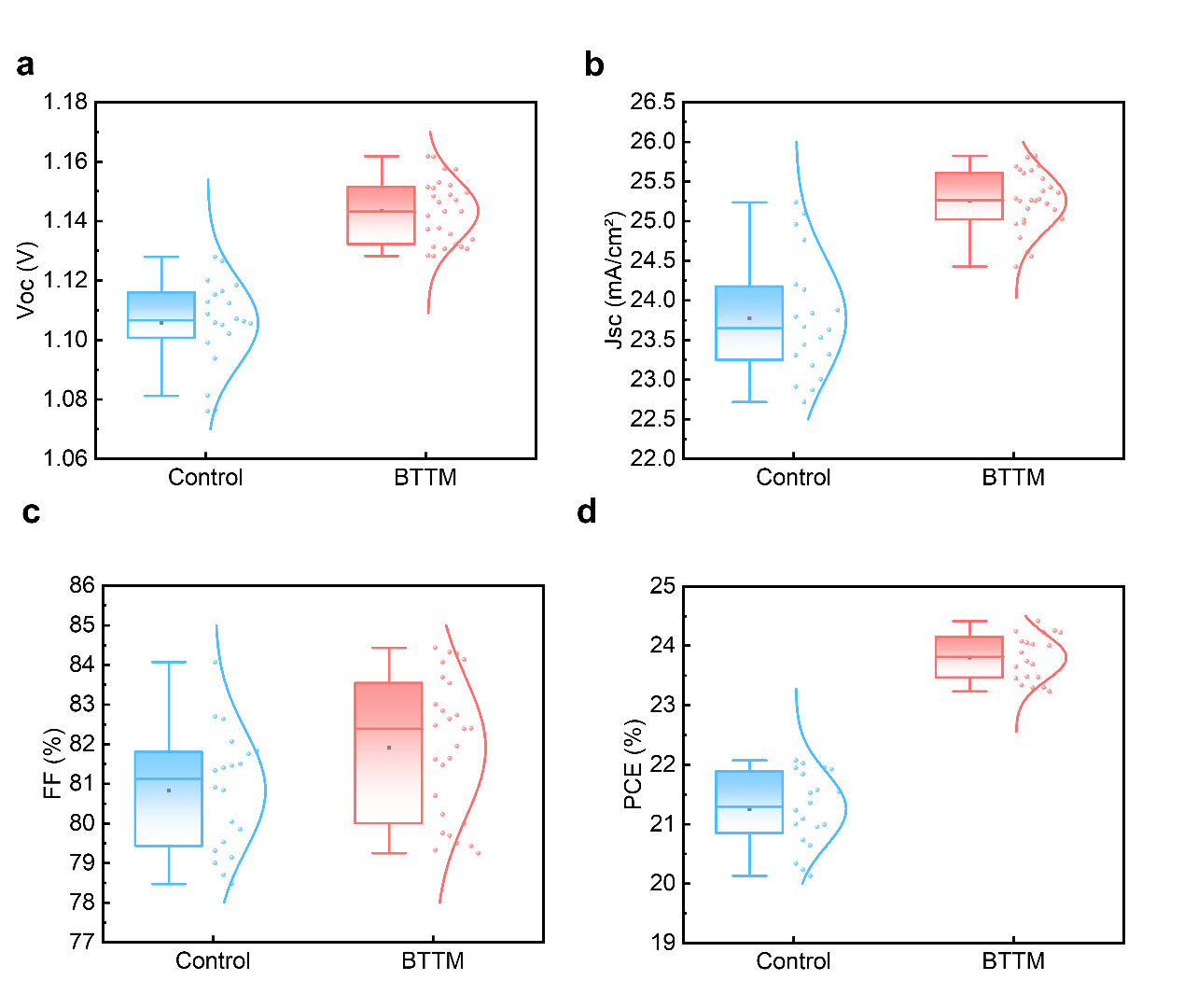


**Fig. S16.** The J-V parameters statistical distribution of Control and optimized BTTM contents devices.

1. V_OC_, b) J_SC_, c) FF, and d) PCE.


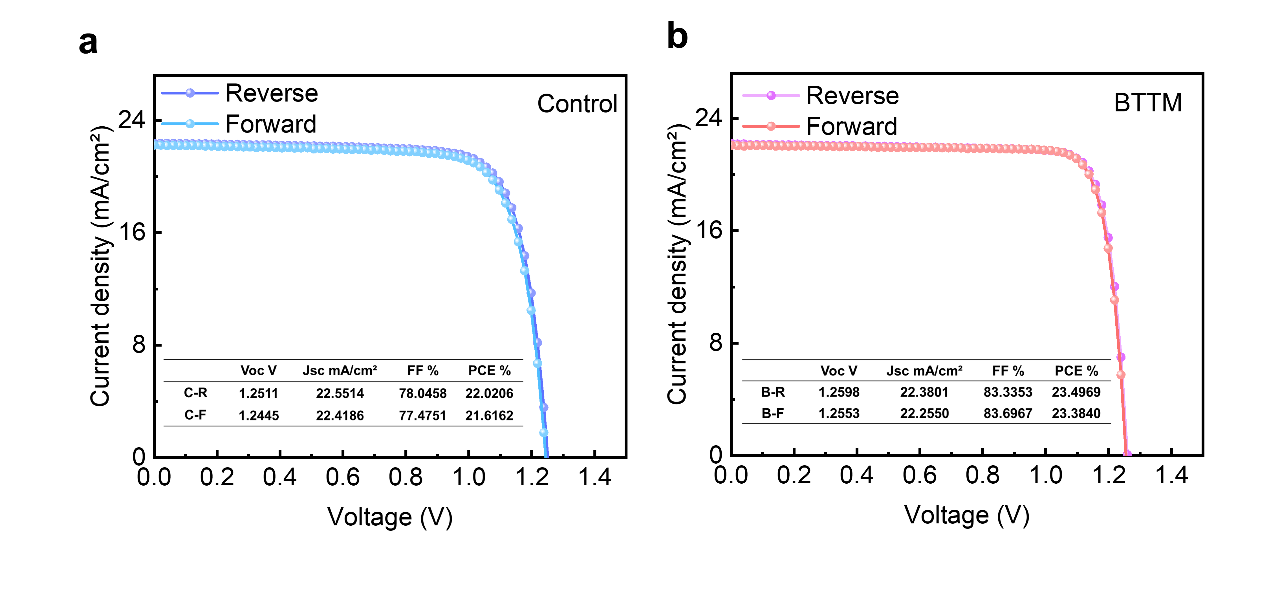


**Fig. S17.** The reverse scan (R) and positive scan (F) J-V curves of the champion WBG PSCs in the Control group (a) and the BTTM group (b).


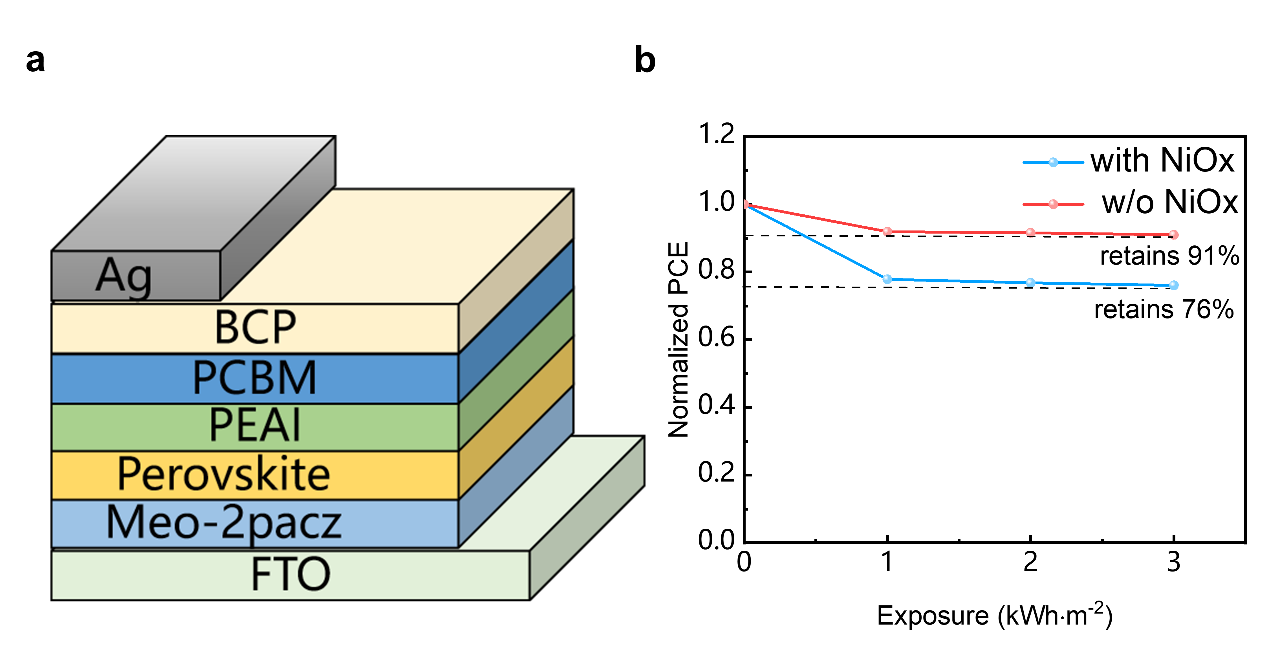


**Fig. S18.** Comparison of ultraviolet stability of devices with and without NiOx layer.

a) Structure diagram of perovskite devices without NiOx layer.

b) PCE comparison chart of devices with and without NiOx layer under ultraviolet irradiation.


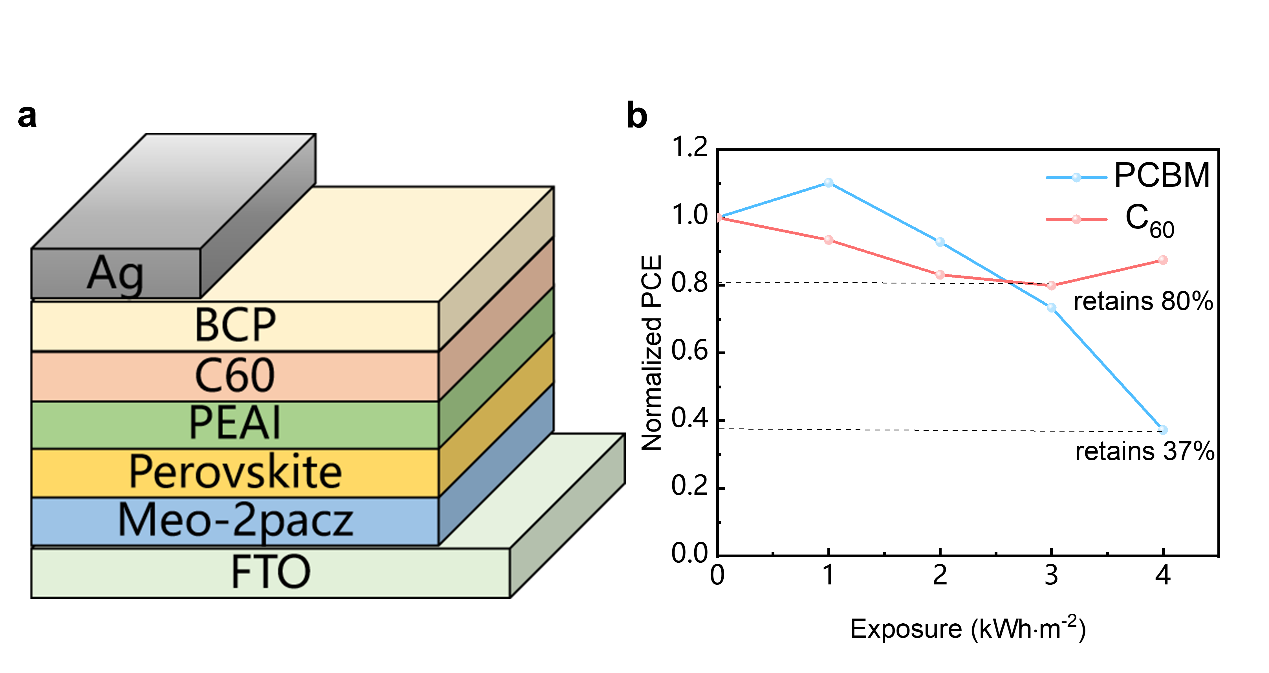


**Fig. S19.** Comparison of ultraviolet stability of devices fabricated by different ETL layers.

a) Structure of perovskite devices.

b) PCE comparison chart of devices in different ETL layers under ultraviolet irradiation.

**Table S1.** TRPL second-order exponential fitting data of perovskite films with different concentrations of BTTM introduced

|  | **A_1_** | **t_1_ /ns** | **A_2_** | **t_2_ /ns** | **t_ave_ /ns** |
| --- | --- | --- | --- | --- | --- |
| 0 wt% | 0.614 | 4.656 | 0.386 | 133.165 | 126.407 |
| 0.05 wt% | 0.639 | 4.161 | 0.361 | 111.925 | 105.261 |
| 0.1 wt% | 0.652 | 4.380 | 0.348 | 348.739 | 340.822 |
| 0.5 wt% | 0.591 | 3.286 | 0.409 | 128.270 | 123.811 |
| 1 wt% | 0.570 | 3.690 | 0.430 | 138.322 | 133.728 |

**Table S2.** TRPL second-order exponential fitting data of Control films and BTTM perovskite films.

|  | **A_1_** | **t_1_ /ns** | **A_2_** | **t_2_ /ns** | **t_ave_ /ns** |
| --- | --- | --- | --- | --- | --- |
| Control | 0.614 | 4.656 | 0.386 | 133.165 | 126.407 |
| BTTM | 0.652 | 4.380 | 0.348 | 348.739 | 340.822 |

**Table S3.** Control device performance and hysteresis index at different scan rates, and R and F represent reverse and forward scans.

| **ID** | **Scan-rate/mV s^-1^** | **V_OC_/V** | **J_SC_/mA cm^-2^** | **FF** | **PCE/%** | **HI** |
| --- | --- | --- | --- | --- | --- | --- |
| C-F | 20 | 1.116 | 24.316 | 78.723 | 21.365 | 0.014 |
| C-R |  | 1.110 | 24.527 | 77.361 | 21.058 |  |
| C-F | 100 | 1.111 | 24.256 | 78.775 | 21.222 | 0.008 |
| C-R |  | 1.106 | 24.397 | 77.962 | 21.042 |  |
| C-F | 200 | 1.109 | 24.242 | 78.265 | 21.036 | 0.009 |
| C-R |  | 1.107 | 24.200 | 77.815 | 20.846 |  |

**Table S4.** BTTM device performance and hysteresis index at different scan rates, and R and F represent reverse and forward scans.

| **ID** | **Scan-rate/mV s^-1^** | **V_OC_/V** | **J_SC_/mA cm^-2^** | **FF/%** | **PCE/%** | **HI** |
| --- | --- | --- | --- | --- | --- | --- |
| B-F | 20 | 1.124 | 25.171 | 82.795 | 23.425 | 0.008 |
| B-R |  | 1.119 | 25.210 | 82.336 | 23.236 |  |
| B-F | 100 | 1.120 | 24.901 | 83.220 | 23.217 | 0.007 |
| B-R |  | 1.117 | 25.105 | 82.143 | 23.041 |  |
| B-F | 200 | 1.122 | 24.881 | 82.419 | 23.012 | 0.001 |
| B-R |  | 1.116 | 24.947 | 82.538 | 22.980 |  |

**Supplementary Note S1.** UV light aging test of perovskite films.

In the UV aging tests conducted in this study, a 365nm LED high-power UV lamp was used for irradiation. Before each irradiation, the irradiance was calibrated using a UV radiometer LH126C, with irradiance levels of 200mW or 100mW. Aging tests of perovskite films were carried out in air. UV aging tests of perovskite devices were conducted in a nitrogen-filled glovebox.
